# Modeling the Evolution of Regulatory Elements by Simultaneous Detection and Alignment with Phylogenetic Pair HMMs

**DOI:** 10.1371/journal.pcbi.1001037

**Published:** 2010-12-16

**Authors:** William H. Majoros, Uwe Ohler

**Affiliations:** 1 Institute for Genome Sciences & Policy, Duke University, Durham, North Carolina, United States of America; 2 Department of Biostatistics & Bioinformatics, Duke University, Durham, North Carolina, United States of America; University of British Columbia, Canada

## Abstract

The computational detection of regulatory elements in DNA is a difficult but important problem impacting our progress in understanding the complex nature of eukaryotic gene regulation. Attempts to utilize cross-species conservation for this task have been hampered both by evolutionary changes of functional sites and poor performance of general-purpose alignment programs when applied to non-coding sequence. We describe a new and flexible framework for modeling binding site evolution in multiple related genomes, based on phylogenetic pair hidden Markov models which explicitly model the gain and loss of binding sites along a phylogeny. We demonstrate the value of this framework for both the alignment of regulatory regions and the inference of precise binding-site locations within those regions. As the underlying formalism is a stochastic, generative model, it can also be used to simulate the evolution of regulatory elements. Our implementation is scalable in terms of numbers of species and sequence lengths and can produce alignments and binding-site predictions with accuracy rivaling or exceeding current systems that specialize in only alignment or only binding-site prediction. We demonstrate the validity and power of various model components on extensive simulations of realistic sequence data and apply a specific model to study *Drosophila* enhancers in as many as ten related genomes and in the presence of gain and loss of binding sites. Different models and modeling assumptions can be easily specified, thus providing an invaluable tool for the exploration of biological hypotheses that can drive improvements in our understanding of the mechanisms and evolution of gene regulation.

## Author Summary

The computational detection of regulatory elements in DNA is a difficult but important problem for decoding eukaryotic gene regulation. Increasing sequence data has made it possible to utilize related genomes, but this is not as straightforward as it may seem, as the evolution of noncoding regulatory regions is relatively poorly understood. In this work we describe a modeling framework and software implementation for aligning multiple DNA sequences to each other while simultaneously predicting functional regions in that DNA (such as the locations where proteins bind to the DNA for the purpose of regulating genes). Those functional regions may or may not be evolutionarily conserved across the sequences. Our framework allows for explicit modeling of evolutionary change across sequences in both the individual nucleotides making up the sequences and in the functional significance of the sequences (functional versus nonfunctional). While most competing frameworks and implementations are limited to a maximum number of sequences and their lengths, ours is scalable. We demonstrate the value of our system by using it to align a set of complex regulatory regions across ten *Drosophila* species and to predict protein-binding sites in those sequences.

## Introduction

A detailed understanding of the mechanisms underlying gene regulation, including precisely how these mechanisms are encoded in an individual's genomic DNA, is of prime importance both to biomedicine and to our knowledge of molecular biology and evolution. Given the wealth of genomic sequence data currently available, computational methods for the modeling and predictive identification of regulatory binding sites play an important role in regulatory genomics. Transcriptional regulation in particular is currently understood in terms of solitary or cooperative binding of enhancer or repressor molecules to key locations in and around target loci. The precise rules governing these binding events and their combinatorial effects on gene expression have been examined in detail for small case studies, but it is currently unknown whether there exists a “regulatory code” or “grammar” that dictates the combination of binding sites, such as number, orientation, distance, and relative spacing.

The use of cross-species conservation in inferring binding-site locations, so-called *phylogenetic footprinting* [1], has become increasingly popular with the greater availability of genomic sequences from related organisms. A major impediment to the use of conservation evidence is the potential for complex evolutionary changes to obscure selection patterns. Though strong purifying selection at the whole-site level is presumed to apply to critical functional sites, the ability for sites to arise, disappear, or be translocated locally within a cis-regulatory module (e.g., via genomic rearrangements or compensatory site turnover, where the loss of binding affinity at one site is accompanied by a compensatory gain of affinity somewhere nearby) renders precise homology relations between sites difficult to establish. In the case of protein-coding genes, the use of pre-computed alignments between syntenic genomic regions often suffices for inference of coding exon boundaries, since the homology of orthologous coding segments is often easily discernible by general-purpose alignment programs. For regulatory binding sites, it becomes necessary to distinguish between *molecular homology*, i.e., common molecular ancestry as defined through DNA replication events, and so-called *functional homology*, i.e., conserved functionality of sites within larger regulatory regions regardless of the precise location.

Effective utilization of cross-species conservation evidence for binding-site identification therefore requires more sophisticated modeling techniques that take into account the potential for incomplete molecular homology. Sites may be present in one group of species but appear at a different location or be lost altogether in a different clade. In addition, because general-purpose alignment programs have been found to perform relatively poorly in aligning noncoding DNA [2], [3], specialized alignment techniques applicable to regulatory elements are desired. We therefore set out to develop a new and flexible framework which integrates the modeling of binding-site evolutionary dynamics directly into the alignment process, resulting in a significant advance in our ability to identify evolutionarily conserved binding sites—even those exhibiting only partial homology. A number of previous works have addressed individual parts of this problem in isolation. For example, Satija *et al.* [4] incorporated the notion of “fast” versus “slow” evolution into the alignment process, but did not explicitly model positional composition biases in binding sites. He *et al.* [5] explicitly modeled binding sites and their evolutionary “gain” and “loss”, but addressed only the two-species case, in which actual gain and loss patterns cannot be fully disambiguated due to lack of an outgroup. Moses *et al*. [6] addressed the problem of modeling more than two species, but did not allow for evolutionary gain or loss. Ray *et al*. [7] modeled gain and loss among multiple species, but utilized precomputed alignments.

Here we describe the first framework which combines all of these tasks into one process: the modeling of binding site evolution, the modeling of nucleotide substitution (including insertion and deletion), and the modeling of binding site residue preferences (positional composition bias), allowing us to simultaneously produce a multiple-species alignment and a set of binding-site predictions informed by conservation patterns. We use this formalism within MAFIA, a new software system for the inference of functional binding sites. We use simulated genomes to precisely benchmark and validate various model assumptions. We show that MAFIA rivals or exceeds the predictive accuracy of current binding-site prediction programs, as well as the alignment accuracy of state-of-the-art alignment programs, and thus combines the best of both worlds within a flexible and integrated system. Applying the system to known *Drosophila* enhancers showcases specific scenarios in which current existing approaches are misled by the complex arrangement of partially conserved binding sites.

## Results

### Overview of Our Modeling Framework

Our models simultaneously capture information about binding propensities of individual transcription factors, rates of evolutionary gain and loss of binding sites, phylogenetic distances and branching patterns between species, nucleotide insertion and deletion propensities, and the substitution biases within each of the various types of genomic elements that may occur in the input sequences. All of this information is utilized for the purpose of simultaneously aligning and annotating orthologous DNA sequences. In the following, we provide a high-level overview of the general layout and salient features of our approach; a detailed description is provided in the Methods section, and algorithms are given in Text S1.

The underlying framework in our system is based on *phylogenetic pair hidden Markov models*. Hidden Markov models (HMMs) capture nucleotide composition biases and spatial organization patterns within a single sequence (see, e.g., [8], [9]). Pair HMMs (PHMMs) perform this modeling simultaneously for two sequences, and also provide a probabilistic model of the precise nucleotide homology relation between the two sequences. Phylogenetic pair HMMs (PPHMMs) generalize PHMMs further by marginalizing over ancestral sequences, thereby allowing them to be used to align multiple sequences related by a phylogenetic tree.

**Figure 1:**
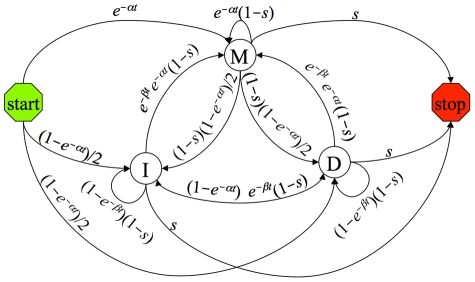
State-transition diagram for a PPHMM implementing a reversible and affine background indel model for a phylogeny branch of length *t*. Ovals denote emitting states; arrows denote transitions. Start and stop are special non-emitting states. This model can be implemented in 33 lines of SEAL code. Parameter *s* = 1-(1- *b*_∞_)(1- *b*(*t*)) gives the probability of leaving the background model, for gain-of-function probability *b*(*t*), *b*_∞_ =  lim*_t_*_→∞_ *b*(*t*). Parameters *α* and *β* influence indel rates.

A PPHMM consists of a set of *states*, which singly or in combination model specific types of genomic elements (such as binding sites for a particular factor), and a set of permissible *transitions* between states, effectively defining a “grammar” governing genomic elements and their preferred spatial relations. Within each state is a full probabilistic model of the genomic element it represents, including: (1) a probability distribution over the nucleotides that can occur at the modeled position, (2) an evolution model describing nucleotide substitution biases, and (3) an insertion-deletion, or “indel” model specifying the propensity for individual nucleotides to be gained or lost along a lineage. All of these probability distributions are automatically scaled according to phylogenetic distances, as denoted by the branch lengths in a phylogeny. Figure 1 shows a state diagram for a simple PPHMM to be used for aligning background sequence; as shown in the figure, the transition probabilities are all functions of branch length *t*.

**Figure 2:**
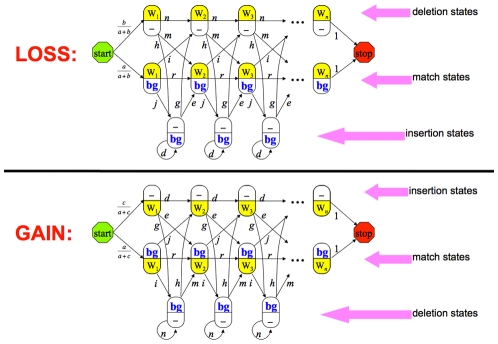
PPHMMs for loss (top) and gain (bottom) of function in a binding site. Ovals are emitting states. The top half of an emitting state denotes the functional class in the parent, while the bottom half denotes the functional class in the child. Dash denotes a gap. *bg* denotes the background functional class. *W_i_* denotes the functional class corresponding to the *i* ^th^ column in a positional weight matrix (PWM). Transition probabilities are derived from the background indel model. Emission probabilities are derived via a substitution mixture model.

To allow for modeling of evolutionary change at the level of site *function*, we define the notion of a *cross-functional state*. Associated with each state are two *functional classes*, one for the ancestral residue, and one for the descendant; each functional class corresponds to a distinct selection regime (i.e., a substitution rate matrix). When the ancestral and descendent classes differ, we say that the state is *cross-functional*, and we model substitution propensities using a mixture model which integrates over all unobservable time points at which the class could have changed along the lineage:
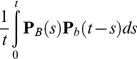
(1)for ancestral class *B* and descendent class *b*, where **P**(*t*) is a substitution matrix scaled to divergence time *t*. PPHMM state diagrams for gain and loss submodels are shown in Figure 2; the ancestral and descendent functional classes are shown in the top and bottom portions of each state, respectively.

**Figure 3:**
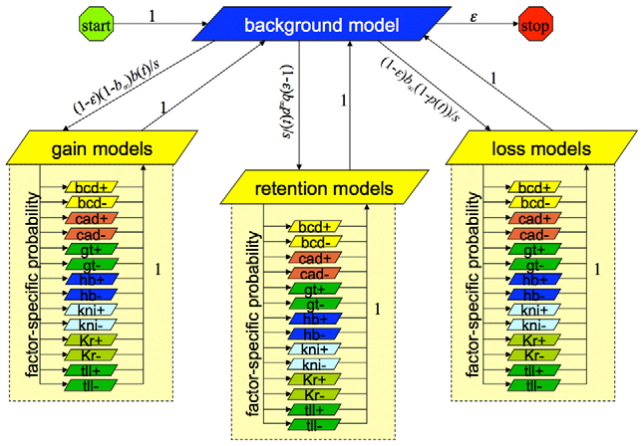
An example CRM evolution model that can be implemented in our framework. Parallelograms denote groups of states in the PPHMM; small parallelograms denote states implementing a binding site profile (positional weight matrix). *b*(*t*): gain probability. *q*(*t*): loss probability. *p*(*t*): retention probability. *b*_∞_: limit of *b*(*t*) as *t*→_∞_. *t*: branch length. *s*: 1-(1- *b*_∞_)(1- *b*(*t*)). *ε*: 0.00001. Plus and minus denote strand. See Materials and Methods for additional details.

Probabilities for entering a cross-functional state are model-dependent (i.e., specified via our model-description language, SEAL), but generally correspond to rates of gain and loss of function. For the experiments described below, in which we account for evolutionary gain and loss of functional binding sites in regulatory regions, we utilized a birth-death process on binding sites for estimating transition probabilities into cross-functional states. Defining *b*(*t*), *p*(*t*), and *q*(*t*) as the probabilities of a functional binding site being born within an interval of length *t*, surviving over such an interval, or dying over such an interval, respectively, we derive a system of differential equations describing the time evolution of these quantities:
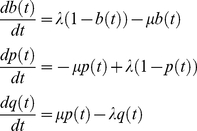
(2)for birth rate *λ* and death rate *µ*. From the solution to these equations we derive transition probabilities for a PPHMM modeling functional binding sites of multiple transcription factors occurring on either strand and subject to stochastic turnover (gain/loss); an example of such a model, for seven transcription factors, is shown in Figure 3.

Performing simultaneous alignment and annotation with a PPHMM such as the one shown in Figure 3 can be accomplished via progressive alignment, optionally followed by some form of *refinement* and/or sampling. Progressive alignment begins with alignment of siblings at the leaves of the phylogenetic tree, and progresses upward. Sibling taxa *T*_1_ and *T*_2_ having sequences *S*_1_ and *S*_2_ can be aligned by finding the most probable *state path* (ordered sequence of states) *φ* ^*^ conditional on the input sequences:
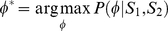
(3)Gaps in the alignment are modeled using the standard insertion/deletion state types as in traditional pair HMMs [8]. Emission probabilities for sibling taxa *Y* and *Z* sharing parent taxon *X* are computed via standard methods used in *phylogenetic HMM*s [10]:
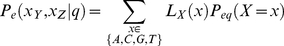
(4)for state *q* and equilibrium distribution *P_eq_*; *L_X_* is a recursive likelihood function which marginalizes over unobservables in the clade rooted at taxon *X*.

In summary, we have augmented standard pair HMMs by both rendering them applicable to unobservable ancestral sequences and by attributing states with *type* information. The latter is achieved by parameterizing state types with distinct substitution models, and by permitting states to employ type-mixture models in the case of evolutionary change-of-function events, as described above. Because standard progressive alignment algorithms are agnostic to specific functional elements and their respective evolutionary propensities, we also devised a novel, two-pass strategy for progressive alignment which takes functional classes into consideration. The aligner first performs a liberal “up-pass” intended to favor sensitivity, followed by a conservative “down-pass” intended to favor specificity. Because functional classes dictate substitution rates and potentially indel propensities during pairwise alignment, this class information can have a potentially large influence on the resulting alignment. To our knowledge, no other multiple-sequence aligner explicitly incorporates such “type” information at a global scale. In the case of cis-regulatory modules, this feature has the potential to reduce the incidence of misalignment between conserved functional elements, including elements that have undergone a gain or loss of function but that still retain at least some degree of identifiable molecular homology.

### Assessing Importance of Different Modeling Features

Our system is implemented as a configurable modeling framework that enables end-users to investigate alternative models for alignment, binding site prediction, and evolutionary reconstruction. As a proof-of-principle, we implemented the model shown in Figure 3 within the context of our modeling framework and applied it to an array of real and simulated data sets to assess the ability of the model to produce both accurate alignments and accurate binding-site predictions. To properly evaluate all aspects, we need to perform such an assessment in the context of sequences in which the “correct” alignment and binding-site annotations are known with certainty. Unfortunately, this is impossible with currently available data sets from real biological systems. Although experimental techniques are available for identifying regions in genomic DNA bound by specific transcription factors, these experiments are typically carried out in only one species, and at this point still suffer from low resolution (i.e., insufficient for precisely delineating the binding sites). As such, predicted binding sites not known *a priori* to be true functional sites cannot with certainty be labeled as false positives. Fine-scaled evolutionary simulations parameterized via measurements taken from real biological data can provide a reasonable approximation to biological reality, while also providing access to the precise nucleotide homology relations and functional elements (e.g., binding sites) produced during the simulation. Currently the most practical approach is thus to supplement real biological data sets with simulations, which allows one to assess sensitivity of site inference on known binding sites, and to assess both sensitivity and specificity in simulated sequences. The utility of simulations for validating models of genome evolution has become increasingly apparent of late [11]–[14].

**Figure 4:**
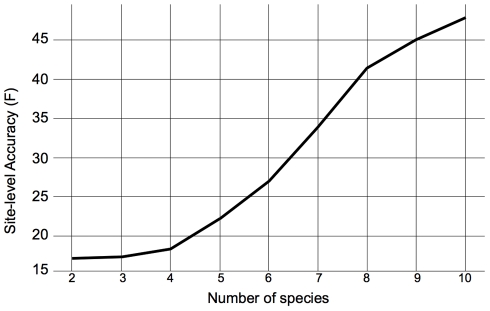
Site-level prediction accuracy as a function of number of species in EVOS simulation runs (the simulator and predictor modeled the same number of species).

To compare alignment and annotation performance under various model assumptions, we first employed two different simulators, both of which allowed binding sites to evolve both at the nucleotide level (via accepted point mutations) and also at the whole-site level (via the gain and loss of site function). The first simulator, EVOS, is based on the same evolutionary model as our aligner; we use it only to explore the impact of changes to the model structure in order to assess the importance of various features in the model. For these simulations we utilized a 10-species *Drosophila* phylogeny: ((((((*melanogaster*, *simulans*), (*yakuba*, *erecta*)), *ananassae*), *pseudoobscura*), *willistoni*), ((*mojavensis*, *virilis*), *grimshawi*)); the model incorporated seven factors: *bicoid* (*bcd*), *caudal* (*cad*), *giant* (*gt*), *hunchback* (*hb*), *knirps* (*kni*), *kruppel* (*kr*), and *tailless* (*tll*). The first modification was to remove gain and loss states from the model, producing what we call the “complete orthology” model. The next modification (called “Phylo-HMM”) employed a simple, three-state PPHMM for alignment, and then performed binding-site prediction by applying the full model (minus gain and loss states) to the root sequence. Note that both of these latter models assume complete orthology during annotation, but only the “complete orthology” model includes states for binding sites during alignment. Prediction accuracy was drastically higher for the full model than for the complete-orthology model when gain and loss events were common, supporting the need for flexible evolutionary models for non-coding sequence analysis. The differences in accuracy between the complete-orthology model and the Phylo-HMM were moderate but consistent, suggesting that the complete-orthology model does derive some advantage from its use of binding-site knowledge during alignment. Figure 4 shows that incorporating additional species did permit the full PPHMM to monotonically increase in accuracy, though the rate of gain decreases starting at nine species. We also applied a “single factors” model which utilized the full PPHMM with gain and loss states but only one of the seven factors used in the simulation; this was repeated for each factor. The single-factors model suffered from low specificity while enjoying high sensitivity, as expected (since this model utilized several runs of the predictor and was therefore less constrained, resulting in an ability to predict overlapping binding sites for different factors). Detailed results obtained with this simulator are given in Text S1.

### Comparing Our System to Earlier Approaches

We now compare the accuracy of our system's binding site predictions to those produced via earlier systems based on simpler models than ours. We first test prediction accuracy on sequences generated by another simulator, *PSPE* [2], that was developed independently of our aligner and which utilizes different modeling assumptions. PSPE allows binding sites to be lost, but only when another site of the same type is gained nearby, thereby modeling strict *compensatory turnover* of functional sites. For the PSPE simulations, we generated 300 root sequences, each 500 bp in length, and then evolved these sequences over a five-species phylogeny: (((*human*, *baboon*), *mouse*), (*dog*, *cow*)); branch lengths were the same as those used by Huang *et al.* [2]. Each sequence was seeded with a single instance of each of six binding sites (factors: MafB, SOX9, IRF1, E2F1, SP1, Sox5); factor weight matrices were obtained from JASPAR [15]. The sequences at the leaves of the phylogeny were then provided to our program, MAFIA, for alignment and annotation of binding sites; an additional ten sequences were evolved and set aside for parameter estimation of the predictor (see Materials and Methods). The average rate of turnover for these simulations was 11.3%; additional statistics are available in Text S1.

To assess relative alignment accuracy, we separately aligned the same sequences using two general-purpose aligners: MUSCLE [16], a relatively recent tool which has been reported to achieve high accuracy on non-coding sequences [2], and CLUSTALW [17], a classic progressive aligner. The alignments produced by the three aligners were compared to the known, correct alignment recorded internally by the simulator during sequence evolution. Alignment accuracy was measured by interpreting an alignment as an undirected graph of homology relations and then comparing the graphs for the correct and predicted alignments; sensitivity (*Sn*) and specificity (*Sp*) of edge prediction were computed and combined into an *F*-score: *F* = 2×*Sn*×*Sp*/(*Sn*+*Sp*). As shown in Table 1, MAFIA's average alignment accuracy was roughly identical to MUSCLE's, while both were noticeably higher than that of CLUSTALW. Note that for this data set, the correct alignments had very few gaps, likely limiting the difficulty of the alignment task; we address this issue in a second set of simulation runs below (see also section 5.1 in Text S1).

**Table 1:** Alignment accuracy for PSPE simulation runs, averaged across runs (CRMs).

program	F	SD
MAFIA	93.2%	1.7
MUSCLE	93.4%	1.9
CLUSTALW	91.0%	2.6

Mean and *SD* values are given for the 300 sequences used in the simulation. *F* = 2×*Sn*×*Sp*/(*Sn*+*Sp*); *Sn* = sensitivity, *Sp* = specificity. *SD* = standard deviation.

To assess relative binding-site prediction accuracy, we compared our program to a well-known comparative binding-site predictor, rMONKEY [6]. Because rMONKEY assumes *complete orthology* of binding sites (i.e., sites do not gain or lose function over evolutionary time), we expected our gain/loss/retention model to produce more accurate predictions on average, since PSPE has the ability to generate gain and loss events. Binding-site predictions were evaluated at both the nucleotide and whole-site levels. At the nucleotide level, individual residues were classified as *foreground* (part of *any* binding site) or *background* (not part of any binding site), and these classifications were scored via the *F*-score. At the whole-site level, an actual binding site was deemed to be found by the predictor if at least half of its nucleotides overlapped a predicted site for the same factor. Note that factor identity was ignored when assessing nucleotide accuracy (e.g., a nucleotide predicted as part of an E2F1 site but that was actually part of an SP1 site was still scored as a success). Nucleotide accuracy thus evaluates the ability of a predictor to detect regions of elevated purifying selection, while the site-level score assesses the ability of the predictor to also identify the correct factor involved.

When scoring the human binding sites only (i.e., ignoring the ability of the programs to identify binding sites in other leaf species), MAFIA outperformed rMONKEY by ∼2.2% at the nucleotide level and ∼2% at the whole-site level (Table 2, *F*-scores only). However, when scoring the programs on all sites in all leaf species, MAFIA's accuracy remained nearly as high as on human only, while rMONKEY's accuracy dropped substantially: 14 percentage points at the nucleotide level and 16 percentage points at the site level. This demonstrates the clear advantage of an integrated approach which is able to reconcile potential conflicts between alignments and binding sites across many species.

**Table 2:** Binding-site prediction accuracy for PSPE simulation runs.

	predict human only						predict all species					
	nucleotide			whole site			nucleotide			whole site		
program	F	Sn	Sp	F	Sn	Sp	F	Sn	Sp	F	Sn	Sp
MAFIA	84.7	82.9	86.6	83.5	81.1	86.2	83.0	79.5	86.9	81.4	77.1	86.3
rMONKEY	82.5	89.0	76.9	81.5	86.5	77.0	68.8	74.5	64.0	65.2	69.1	61.6

Left half of table: accuracy of predictions in human. Right half of table: pooled accuracy over all leaf species. For site-level accuracy, a known site must overlap a predicted site of the same factor by at least half its nucleotides to be counted correct. All numbers are percentages, averaged over 300 simulation runs.

For a second comparison, we utilized 142 sequences originating from the RedFly database [18], which is derived primarily from DNAse I footprinting experiments. Only *D. melanogaster* sites are annotated in this set of “known” sites. Benchmark evaluations such as this can only assess annotation accuracy on one “target” species, which addresses only one (and not the crucial) aspect of our modeling approach. Nevertheless, this exercise will serve to show that our model extends the possibilities to predictions in multiple genomes, while achieving competitive performance on the simpler single-genome task. We chose to address the simple scenario of predicting individual sites within a CRM. In previous investigations [5] authors have typically constructed a “gold standard” set of sites by identifying the highest-scoring positional weight matrix (“PWM”) position in each footprinting region and taking that as the “known” site for each footprint. This is doubly problematic and has the potential for circularity: for one, the PWMs are often created from the very footprinting sites used as the gold standard, and secondly, the same PWM parameters are usually employed within the model itself. We therefore evaluated sensitivity of predictions by observing whether each predicted site overlapped a DNAse I footprint for the same factor, and then separately evaluated false positive rate by counting predictions in “decoy” CRMs as false positives. In these experiments MAFIA was found to have similar performance improvements compared to rMONKEY as we observed above; details are given in Text S1.

### Assessing Predictions in Complex Regulatory Regions

To evaluate the value of our approach for the analysis of real, complex, and well-studied CRMs, we ran MAFIA on a set of developmental enhancers from the early embryo segmentation network, which has been frequently utilized as a benchmark. We used seventeen enhancers as annotated by He *et al.* [5], and used the binding site models provided by those authors. Because the footprints in RedFly are often larger than the actual binding sites, annotations were based on scanning the footprints with a PWM to identify precise boundaries for the putative binding site. As mentioned above, this process likely induces some biases.

We evaluated our program MAFIA running the same model described above, as well as the programs rMONKEY, EMMA [5], and PhyloGibbs-MP [19], as to their ability to identify these putative binding sites, with *D. melanogaster* once again chosen as the target species (since binding site information is highly incomplete in other genomes). EMMA is the more similar of the programs to ours, since it is based on an explicit model of gain and loss and aligns the sequences within a binding-site-aware framework; however, it is currently limited to two species. We therefore performed three sets of experiments, corresponding to three phylogenies: a two-way phylogeny, (*melanogaster*, *pseudoobscura*), a six-way phylogeny, ((((*melanogaster*, (*yakuba*, *erecta*)), *ananassae*), *pseudoobscura*), *virilis*), and a 10-way phylogeny, ((((((*melanogaster*, *simulans*), (*yakuba*, *erecta*)), *ananassae*), *pseudoobscura*), *willistoni*), ((*mojavensis*, *virilis*), *grimshawi*)). We employed 17-way cross-validation—i.e., training each model on 16 sequences and testing on the remaining sequence. Training of EMMA was carried out via software included in the EMMA software distribution (program “weight_est”). Training of MAFIA was by simple hill-climbing on individual parameters (see Materials and Methods). Training of rMONKEY was by simple hill-climbing on the single *P*-value threshold used to filter predictions. In all cases, the objective function for training was site-level prediction accuracy on the training sequences. The program PhyloGibbs-MP is a *de novo* motif finder but also accepts matrix files specifying known motifs and will predict sites based on conservation evidence; this is how the program was used here.

When performing prediction based on only two species, MAFIA substantially outperformed EMMA in its recommended configuration (Table 3). Because EMMA can utilize only two input sequences at a time, our 6-way comparisons included only MAFIA and rMONKEY. MAFIA's accuracy was again superior at the whole-site level (by 2.4%). MAFIA was also applied to the 10-species set, as was PhyloGibbs-MP. rMONKEY was unable to evaluate all 10-way alignments, as some enhancers resulted in memory consumption of over 8 GB. MAFIA applied to 10 species produced the best nucleotide-level accuracy observed, and the second-best site-level accuracy (second to the 2-way MAFIA run), demonstrating that the incorporation of additional informant sequences can improve annotation accuracy for a single target genome, but need not do so in all cases. In addition, the insight gained from a comparison between runs which differ in the number of species used is inherently limited, as the information gained from the ability to annotate additional related genomes is not reflected. Note also that the nucleotide accuracy scores do not reflect the ability to correctly identify the specific factor associated with a binding site, which is a key goal of our system.

**Table 3:** Site-prediction accuracy on 17 *Drosophila* developmental enhancers.

		nucleotides			whole sites		
program	#taxa	F	Sn	Sp	F	Sn	Sp
MAFIA	10	*48.2*	51.4	45.4	*41.3*	42.0	40.6
rMONKEY	10	-	-	-	-	-	-
PhyloGibbs-MP	10	37.9	94.0	23.7	15.5	71.5	8.7
MAFIA	6	44.5	40.6	49.1	*41.0*	36.1	47.3
rMONKEY	6	*45.6*	50.4	41.7	38.6	40.8	36.7
MAFIA	2	*46.7*	43.9	49.8	*42.3*	38.5	46.8
EMMA	2	33.6	71.3	22.0	22.3	45.0	14.8
EMMA-E	2	47.1	62.6	37.8	37.3	46.2	31.2

Results from rMONKEY on the 10-way alignments are missing because the program does not process the longer sequences due to large (>8 GB) memory requirements. All numbers are percentages. [“EMMA-E” results were obtained by running EMMA in a nonstandard configuration (command line option “-e”), which forces prior densities to be re-estimated on the test sequence. We included them here for completeness as they led to a noticeable improvement at least on this set of enhancers.]

When applied to 10 species, MAFIA took 12.5 minutes on average (SD: 10.5, max: 36.9) per CRM when using 8 CPU cores, and 304 MB of RAM (SD: 65.5, max: 453). The 10-species data files contained 600 bp sequences on average (SD: 450, max: 2400). Note that increasing the number of species to be aligned results in only a linear increase in computational complexity within our system, while our use of the Hirschberg algorithm [20] permits the application of very large models without incurring exorbitant memory costs.

It is worth noting that the test set includes cases of overlapping binding sites, which are indeed known to occur, either due to functional reasons (such as mutual steric occlusion of activators and repressors—e.g., [21]) or fortuitously constrained co-evolution [11]. Though currently MAFIA cannot produce overlapping predictions, MAFIA predictions for one factor sometimes overlapped a known site for a different factor; these are counted as false positives in our evaluation, though for pairs of factors known to commonly overlap these may instead indicate true sites that are missing from the “gold standard”. Two such cases are *kruppel* overlapping *bicoid*, and *giant* overlapping *bicoid*; these overlapping pairs are widely known to function as competitive repressor-activator sites [22], and are present both among the known sites and in the overlaps between MAFIA predictions and known sites (see tables S2 and S3 in Text S1). Although MAFIA currently lacks the ability to predict overlapping binding sites (except via separate runs with different factors), the future incorporation of a sampling procedure should permit the independent prediction of binding site instances via estimates of posterior probabilities.

**Figure 5:**
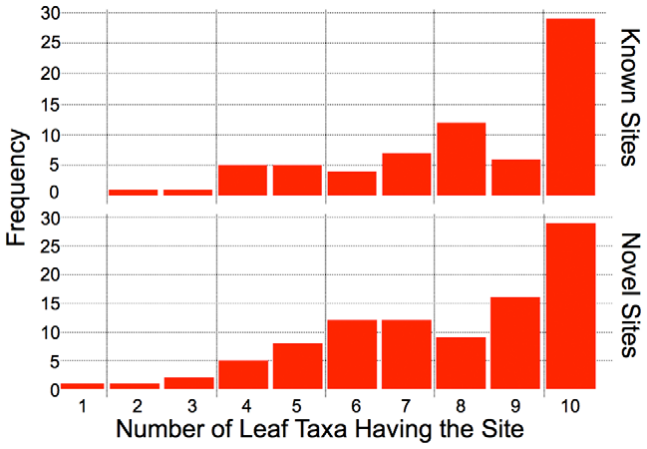
Histogram of number of extant *Drosophilids* predicted to share a given site, for known sites (top pane) and novel predicted sites (bottom pane), over a 10-way phylogeny.

In addition to identifying many known sites, MAFIA predicted a number of novel sites in this test set. As shown in Figure 5, the known and novel sites in *D. melanogaster* had very similar degree-of-orthology distributions, with a preponderance of well-conserved sites in both cases. This suggests that many of these novel sites are governed by similar selection pressures and contribute to the function of the enhancer.

### Assessing Predictions Using a Novel ChIP-Seq Analysis

**Figure 6:**
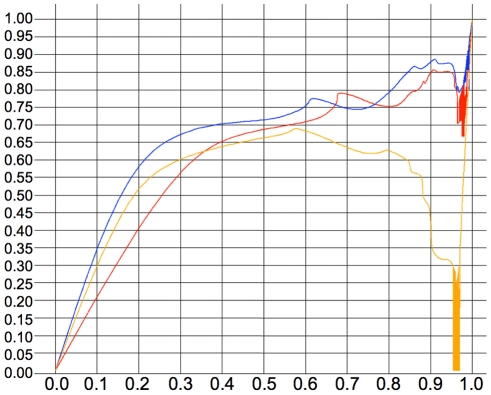
ROC-like curve for MAFIA (blue) applied to ten species, rMonkey (red) applied to six species, and the “gold standard” (gold). Sensitivity is plotted on the y-axis, false-positive rate along the x-axis. Each point corresponds to a different stringency threshold in the processed ChIP-seq data.

The analysis of “gold standard” binding-site annotations followed the example of previous studies, but others have recently pointed out the presence of inherent biases in such datasets (incompleteness and presence of weak but conserved, or partially conserved, sites) which impede the evaluations of multi-genome methods [23]. We therefore evaluated the same 17 enhancers via a novel evaluation strategy which utilized genome-wide chromatin immunoprecipitation (“ChIP-seq”) data obtained from Bradley *et al.* [24] to construct an ROC-like curve. Figure 6 plots *sensitivity* (TP/(TP+FN)) on the *y*-axis and *false-positive rate* (FP/(FP+TN)) on the *x*-axis, for a large ensemble of peak-calling thresholds (see Materials and Methods). MAFIA (blue) has a significantly larger area under the curve (AUC) than rMONKEY (red): .67 versus .59 (Wilcoxon *P* = 2.2×10^−16^). The “gold standard” (gold curve) gives an AUC of only .54, showing that this set of “known sites” does indeed omit many elements that are supported by ChIP-seq data and that both MAFIA and rMONKEY are able to find using only conservation evidence.

**Figure 7:**
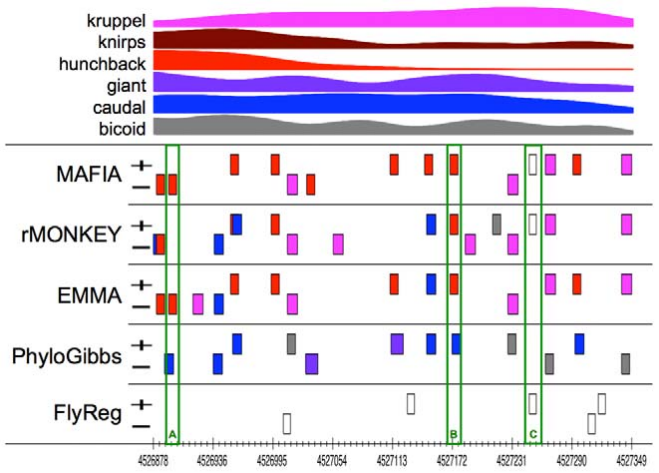
An example *D. melanogaster* developmental enhancer. At top are F-Seq scores from ChIP-seq data for six transcription factors (kr = *kruppel*, kni = *knirps*, hb = *hunchback*, gt = *giant*, cad = *caudal*, bcd = *bicoid*); curves were scaled to maximize visual impact for the figure. Predictions and known sites are shown below, with colors denoting factor identity as per the F-Seq curves (factor *tailless* was not assayed in the ChIP-seq experiments and is shown in white). Plus and minus tracks correspond respectively to sense and antisense strands of the dm3 assembly for chromosome 3R. The FlyReg track depicts known binding sites according to the “gold standard” (see text). The EMMA track was produced using the –e option for that program.

**Figure 8:**
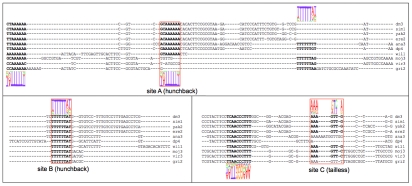
Example MAFIA alignments from the CRM shown in **Figure 7**. Nucleotides predicted to participate in binding are shown in bold. Weight matrices for factors are shown as sequence logos above (sense strand) or below (antisense strand) the alignment.

While this evaluation clearly shows the overall advantage of our method, it is instructive to examine individual enhancers to illustrate the advantages of MAFIA in more detail. Figure 7 depicts a sample CRM including known sites from the “gold standard”, predictions from several programs, and density scores (F-Seq [25]) obtained from the genome-wide ChIP-seq data. For this particular CRM, the gold standard had only four sites annotated, all putative *tailless* sites (colored white, since this factor was not included in the ChIP-seq assay). This enhancer is a striking case for the incompleteness of the gold standard, as it is immediately noticeable that many predicted sites from different programs clearly fall close to a peak in the corresponding density curve. Though not all predicted sites fall near a peak, several non-peak sites were agreed upon by multiple predictors, lending support to their functional validity (see below). Figure 8 shows the MAFIA alignments and annotations for a number of interesting sites (green bars), which we highlight in the following.

Site A (*hunchback*) is strongly supported by ChIP-seq data, but the clear lack of full conservation beyond the *melanogaster+obscura* groups renders this site difficult to predict for some programs. Though rMONKEY was able to predict the well-conserved *hunchback* site shown at the left edge of the alignment, it was unable to predict site A. EMMA was fortuitous in being able to predict the site, since the site is fairly well conserved between *D. melanogaster* and *D. pseudoobscura* (the two species used for the EMMA runs); it would likely have missed the site if the second species had been chosen from outside the *melanogaster+obscura* clade.

Of particular interest is the presence of a possible hunchback site a short distance 3′ of site A which appears to be absent from the *melanogaster* subgroup but present in most species outside this clade. Note that these two sites reside on opposite strands, precluding the possibility that these are simply homologous sites that have been mis-aligned. Parsimony considerations would suggest that the forward-strand site is an ancient element that was lost in the *melanogaster* subgroup (as well as along the lineage leading to *D. willistoni*), while site A was gained somewhat earlier than this loss event, on the common lineage leading to the *melanogaster* and *obscura* groups. Under this hypothesis, the existence of site A may have reduced the strength of selection maintaining the forward-strand element near site A in the *melanogaster* subgroup, leading to the latter site's demise. The very possibility for these types of context-dependent turnover events demonstrates a potential for further extensions to models of evolutionary patterns in conservation-based prediction systems.

Site B is an example of a prediction with minimal support from the ChIP-seq data, but which is very likely a functional binding site nonetheless. This putative *hunchback* site is perfectly conserved across all ten species, while flanking sequences show less-than-perfect conservation, suggesting strong purifying selection specific to the eight positions making up the putative site. The consensus sequence perfectly matches that of the weight matrix for this factor, and produces a likelihood ratio of 5.4 when evaluated using the weight matrix with a 2^nd^ order Markov chain as background. Three of the four predictors (MAFIA, rMONKEY, and EMMA) agree in placing a *hunchback* site at this precise location. Assuming that the site is indeed functional within some spatiotemporal context in the organism's lifecycle, this example illustrates the limitations of ChIP-seq data for establishing the validity of putative binding sites. In this particular case, whole embryos were collected during a narrow window of time corresponding to the anterior-posterior pattern formation stage of *Drosophilid* embryogenesis [24]. The transcription factors assayed are well known to play prominent roles in anterior-posterior pattern formation, but this does not preclude other uses of these factors later or earlier in the organism's lifecycle. Spatial resolution may also play a role in obscuring binding affinity for particular sites, since the use of whole embryos will bias the results toward more obligately bound sites, potentially leaving the signal from lower-bound sites, or sites bound in small numbers of cells, below the effective noise threshold.

Finally, site C (*tailless*) demonstrates the ability of our system to predict sites that are not only not conserved in other species, but that may be interrupted in the alignment by indels; most phylo-HMM based methods will miss sites such as these because they prohibit indels within binding sites. Though rMONKEY did predict a conserved site in the 6-way alignment at this location, in order to do so it was forced to predict consensus sequences for *D. pseudoobscura* (ACAATCT) and *D. ananassae* (AATGTCT) that are relatively poor matches to the weight matrix for this factor.

## Discussion

A variety of methods have been explored in recent years for alignment of DNA sequence, for identification of functional binding sites, and, in relatively few cases, for performing both simultaneously. The incorporation of binding-sites into the alignment model is an attractive idea, since the presumed higher level of conservation of binding sites in many instances may aid the alignment process by providing “alignment anchors”, which in turn should ease the task of identifying binding sites in the resulting alignment (based on conservation patterns).

Several recent studies have proposed techniques for adapting pair HMMs to the problem of multiple-sequence alignment, specifically within the context of regulatory modules. The work presented here continues in a direction similar to these latter efforts, but provides a more thorough computational foundation for detailed models of regulatory sequences and their evolution. The two key aspects of this foundation are: (1) the ability to implement different sequence evolution models specified via a simple but powerful modeling language; and (2) the sole reliance on pairwise sequence models (PPHMMs), which permits the use of far larger numbers of functional classes than would be possible via, e.g., composing transducers (as in [26]) into *N*-ary HMMs, for large *N*. We showed that our implementation's alignment accuracy and its success at binding site identification is competitive with current state-of-the-art tools for either problem, while scaling to allow for the evolutionary analysis of multiple genomes (e.g. ten *Drosophila* species).

Explicit modeling of evolutionary (non-compensatory) turnover in regulatory binding sites has recently seen an increase in interest, both in the two-species case [5] and for larger numbers of species [7]. The propensity for regulatory sequences to experience significant evolutionary change, including wholesale rearrangement of binding sites, has been well documented [27], [6], [28]. Incorporating turnover into predictive models is nontrivial, however. As noted by Hawkins *et al.* [23], relaxation of the “complete orthology” assumption effectively increases the difficulty of the classification task (site vs. non-site) because it decreases the difference between the classes to be distinguished (i.e., it decreases the classifier's achievable “margin”). Incorporation of a gain and/or loss mechanism in a binding site model should therefore be expected to improve sensitivity at the expense of specificity. In our own simulation runs (EVOS data set #1—see Text S1), we found precisely the opposite, with the overall accuracy (*F*-score) improving after the incorporation of gain and loss states in our model, with the improvement being rather drastic when high levels of turnover were present in at least some of the test sequences. This disagreement with the theoretical findings of Hawkins *et al.* may be due to the effect of the gain and loss states during the alignment process; in the work by Hawkins *et al.*, the alignments were pre-computed by a general-purpose aligner with no knowledge of binding sites. He *et al.* [5], who utilized gain and loss states during the alignment, also found an improvement in prediction accuracy when gain and loss states were enabled. Ray *et al.* [7], who modeled gain and loss in a multiple-species setting but relied on pre-computed alignments, also found that their model outperformed other systems lacking gain and loss states, and that this advantage was relatively stable across different rates of turnover in simulated data.

Another interesting result of the Hawkins *et al.* [5] study was their conclusion that “gold standard” test sets tend to be biased against methods capable of detecting weak binding sites (due to the experimental protocols involved in identifying the sites in the gold standard), and are therefore biased against models such as Phylo-HMMs which can detect sites with weak binding profiles but strong conservation. The 17-CRM *Drosophila* data set used here likely suffers from another obvious confounding problem: annotated sites were identified by scanning footprinted sequence data with a simple weight matrix and calling the highest-scoring interval the “correct” site [5], which will bias annotations toward strong sites in one annotated target species, while also being incomplete. Our use of ChIP-seq data to construct ROC-like curves provides a more unbiased means for evaluating the relative merits of competing prediction systems. Whereas other authors have applied peak-calling algorithms to ChIP data to identify putative sites, our procedure mitigates the uncertainty inherent in the peak-calling process by identifying putative binding regions at many different binding thresholds. The resulting ensemble of nested peaks is used to compute a set of sensitivity × false-positive-rate pairs that can be compared both visually, via an ROC-like curve, and more rigorously via formal statistical tests. As ChIP data continues to become available in larger volumes and for larger numbers of organisms, we believe this type of analysis will become increasingly valuable for those investigating computational methods for binding-site prediction.

In summary, it has been shown here that in settings in which we know the actual evolutionary history of sequences (via model-independent simulations) or can make use of genome-wide direct binding evidence (rather than a manually annotated “gold standard”), our modeling framework shows strong improvement in predictive capacity over previous attempts that relied on precomputed alignments, were limited to small numbers of sequences, or made untenable simplifying assumptions (such as complete orthology). We have concentrated here on the ability to accurately predict functional binding sites, but our framework also provides a means to align sequences as well as simulate the evolution of sequences and functional elements in those sequences, at both the nucleotide and whole-element level. In particular, it is straightforward to impose “grammatical” restrictions such as relative order or orientation of sites. We expect that the flexibility of this framework will allow further improvements to predictive accuracy as a wider range of models are investigated within the context of this easily configurable system.

## Materials and Methods

### Data

The set of seventeen Drosophila CRMs consisted of developmental enhancers previously utilized by He *et al.* [5]; several enhancers from the original set were excluded from our analyses because they either contained no instances of binding sites for the factors included in this analysis, or they caused one of the external software packages in our comparison to malfunction. Because the footprints in RedFly are often larger than the actual binding sites, the latter authors scanned the footprints with a positional weight matrix to identify precise boundaries for the putative binding site. This process likely induces a bias in favor of prediction methods incorporating a weight-matrix-like approach. The CRMs had a mean length of 612 bp (range: 67–1889), totaling 62391 bp (10028 in *D. melanogaster* alone).

The 142 RedFly footprinting profiles had a mean length of 662 bp (range: 610–1908), totaling 564345 bp (87824 in *D. melanogaster* alone); the “decoy” CRMs had a mean length of 495 bp (range: 500–5114), totaling 421644 bp (71000 bp in *D. melanogaster* alone). The decoy sites were chosen to have the same G+C density (within one half percent) and the same PhastCons [29] conservation level (these levels are given in 10% increments when downloaded from the UCSC [30] genome browser) as one of the true CRMs (a different such CRM for each decoy site).

For the PSPE simulation runs, we parameterized the simulator identically to Huang, *et al.* [2]—i.e., with a Markov order of 3, a negative Binomial gap model (with parameters 1 and 0.5), an HKY substitution model (with parameter 0.05), a *gamma* value of 1, an *iota* value of 0.1, and a *lambda* value of 0.1. Sequences had a mean length of 500 bp (range: 475–530), totaling 274975 bp (54927 bp for *D. melanogaster* alone).

For the EVOS simulation runs, the mean CRM length was 503 bp (range: 71–877), totaling 1046498 bp (104735 for *D. melanogaster* alone).

### Models

A *phylogenetic pair hidden Markov model* (PPHMM) *M* = (*Q*,*A*,*P_t_*,*S*) consists of a set of states *Q*, an alphabet *A* which we take here to be the nucleotide alphabet, a state transition function *P_t_*(*q_j_*|*q_i_*) giving the probability of transitioning from state *q_i_* to *q_j_*, and a conditional substitution matrix *S* giving the probability of one symbol from *A* being substituted by another symbol from *A*, conditional on state *q*: *P*(*a*_2_|*a*_1_,*q*). A PPHMM can be instantiated from a PPHMM template *T* = (*Q*,*A*,*C_t_*,*R*), where *Q* and *A* are as defined above, *C_t_* is a set of *closures C_t_*(*q_j_*|*q_i_*,*t*) giving the probability of transitioning from state *q_i_* to *q_j_* conditional on a time parameter *t*, and conditional substitution rate matrix *R* gives the instantaneous substitution rates between symbols in *A* (conditional on state). Given a branch of length *t* in a phylogeny, a PPHMM for that branch can be instantiated via *M* = (*Q*,*A*,*C_t_*(*t*),*e^tR^*), where *C_t_*(*t*) denotes the function *P_t_*(*q_j_*|*q_i_*) = *C_t_*(*q_j_*|*q_i_*,*t*). PPHMM templates can be specified compactly in our system using the *SEAL* modeling language, as described in Text S1.

For the experiments reported here, we implemented binding-site profiles (for “retention” events—i.e., no gain or loss of function) via a linear sequence of PPHMM states trained via the Halpern-Bruno construction [31] as applied to a JASPAR matrix. (The Halpern-Bruno construction provides a means of obtaining a substitution rate matrix for a foreground class, given a reversible background substitution rate matrix and a foreground equilibrium distribution). Phylogenies were constructed via the neighbor-joining algorithm [32]. Pre-constructed alignments used for training MAFIA and as input to competing programs were built using MUSCLE. All substitution rate matrices were general time-reversible models. Training of rate matrices was carried out as described previously [33].

Our background model for these experiments (Figure 1) consisted of three states (an *insert*, *match*, and *delete* state) trained from the MUSCLE training alignments. The model is affine, since the probability of a transition from the match state to an indel state can differ from the self-transition probabilities within the indel states. The model is also *reversible* (or *symmetric*) in the sense that identical alignments are produced whether sequence A is aligned to B or B is aligned to A; many popular models (e.g., TKF91—[34]; SPH08—[4]) do not have this property. The probability *s* = *s*(*t*) of leaving the background model is given by *s*(*t*) = 1-(1- *b*_∞_)(1- *b*(*t*)), for *b*_∞_ = lim*_t_*_→∞_ *b*(*t*), where *b*(*t*) is the *gain probability* for binding sites (to be defined shortly); *s*(*t*) dictates the density of binding sites. *α* and *β* are parameters to the model; their relative values influence the frequency and average lengths of gaps.

Binding-site gain and loss events were modeled using PPHMMs of the form depicted in Figure 2. The top half of each state in the figure is labeled with the functional class of the ancestral residue, while the bottom half is labeled with the class of the descendent residue; *bg* represents the background functional class, *W_i_* represents the functional class for the *i* ^th^ column of the corresponding binding profile, and a dash indicates a gap rather than a residue, in the case of insertion and deletion states. (For retention states the ancestral and descendent functional classes would be identical). Transition probabilities (denoted *a*, *b*, *c*, …, *n* in the figure) are derived from the background model (see Figure S1 in Text S1). This model enforces the constraint that indels are not permitted inside functional binding sites, but permits them to occur in non-functional sequences orthologous to functional binding sites.

An example illustrating the utility of these indel states in the gain/loss submodels is given in Figures S4 and S5 in Text S1. As shown in the figure it is quite possible to have strong matches to a binding site profile in several organisms while one or more other organisms show clear nucleotide homology with those sites without retaining a strong match to the binding site profile. Without indel states in the gain/loss submodels, these (putatively) orthologous non-functional nucleotides would often be incapable of aligning with the putative binding sites in the other sequences, and would contribute more gaps to the alignment.

The background, retention, gain, and loss submodels were merged into a single PPHMM template which was then used to instantiate the models used for the computational experiments. Because transitions between submodels were constrained for these experiments to be between the background submodel and a foreground (gain/loss/retention) submodel (see Figure 3), transitions between submodels were governed by the probabilities for gain, loss, and retention events on binding sites; these are described next.

Gain, loss, and retention events were modeled via a stochastic birth-death process, as follows. Let *A* be an ancestral taxon having a descendant *D*, with *D* following *A* by *t* time units. For any binding site present in *A*'s genome, *p*(*t*) will denote the probability that the orthologous site in *D* is a functional binding site (for the same factor), while *q*(*t*) will denote the probability that the site is not functional in *D*. We model the time evolution of *p*(*t*) and *q*(*t*) via the following set of differential equations:
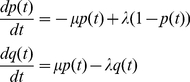
(5)with initial conditions *q*(0) = 0 and *p*(0) = 1; parameter *λ* is the instantaneous *birth rate*, while *µ* is the instantaneous *death rate*. The above system admits the following solution:
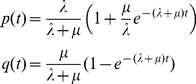
(6)For any vacant interval of the proper size in the ancestral genome (lacking a binding site of any kind), we denote by *b*(*t*) the probability that the orthologous site in the descendant will now be a functional binding site for some transcription factor:
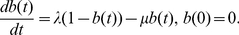
(7)This equation admits the following solution:
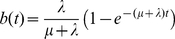
(8)The limit of this term, lim*_t_*_→∞_ *b*(*t*) = *b*_∞_ = *λ*/(*λ*+*µ*), provides the probability for leaving the background state. The probabilities of entering a gain, loss, or retention submodel are derived from *b*(*t*), *q*(*t*), and *p*(*t*), respectively. We call *b*(*t*) the *gain probability*, *q*(*t*) the *loss probability*, and *p*(*t*) the *retention probability*.

Evolutionary rates of individual nucleotides in gain and loss states are assessed in MAFIA via a mixture model combining the foreground and background substitution rates. Let *B* and *b* denote two different functional classes, *B* for the ancestor and *b* for the descendant. **P** *_B_*(*t*) and **P** *_b_*(*t*) denote the respective substitution matrices for these classes. The mixture substitution model **P** *_B_*_→*b*_(*t*) is given by:
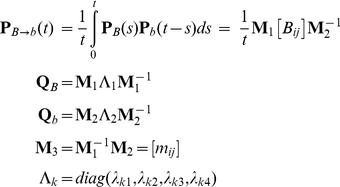
(9)where **M** *_i_* and **M** *_i_* ^−^ ^1^ are found via spectral decomposition of **Q** *_b_* or **Q** *_B_* (the instantaneous rate matrices from which the corresponding **P**(*t*) matrices are derived), and *B_ij_* is given by:
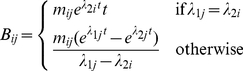
(10)for *λ_kj_* the eigenvalues resulting from the spectral decompositions. **P** *_B_*_→*b*_(*t*) is used in assessing substitution probabilities in any cross-functional state having ancestral functional class *B* and descendent class *b* (*B*≠*b*).

### Algorithms

MAFIA performs multiple-sequence alignment using a bottom-up progressive alignment approach followed by a top-down refinement step, as described in the Results. During the bottom-up progressive phase, a modified version of Felsenstein's algorithm [35], which we call *Lossy Felsenstein*, is used; this algorithm permits retention and loss events, but not gain events, in order to promote sensitivity and reduce greedy behavior. Once the progressive up-pass is complete, we apply Dollo parsimony [36] in an attempt to filter spurious ancestral nucleotides that were too liberally propagated up the tree during the up-pass; Dollo parsimony effectively finds the clades where individual features were first introduced by evolution (according to a parsimony criterion). Finally, we apply a down-pass to remove any inconsistencies introduced by the Dollo procedure.

During the up-pass, each pair of sibling taxa in the phylogeny are aligned by performing Viterbi decoding [37]; emission probabilities are computed via Felsenstein pruning, as previously noted. The PPHMM for this sibling-alignment step is obtained by instantiating the PPHMM template on a branch of length *t*_1_+*t*_2_, where *t*_1_ is the branch length between the left sibling and its parent in the tree, and *t*_2_ is the length of the branch between the right sibling and its parent. This step differs from the sibling alignment step in the approach of Holmes and Bruno [26], who instead compose the transducers on the two sibling branches into a single “triple HMM” with an enlarged state space. Decoding of the triple HMM will produce a pair of alignments: one between each sibling and its parent. Composing pair HMMs with hundreds of states each into a triple HMM would result in a vastly larger state space, so we instead construct the PPHMM for aligning the siblings directly, as previously stated. This results in an alignment *A_LR_* between siblings *L* and *R*. In order to obtain alignments *A_PL_* and *A_PR_* between parent *P* and each sibling, we assume (only during the up-pass) that all residues in either sibling are present in the parent; this produces unambiguous alignments between the parent and each sibling, while retaining a similar flavor to the Lossy Felsenstein algorithm described above (since it permits only deletions and matches during the up-pass). Though this strategy favors increasing sequence lengths for taxa higher in the tree, the Dollo parsimony procedure performed after the up-pass reduces sequence lengths by identifying clades where (according to the Dollo principle) a nucleotide should be considered to have originated.

During the down-pass, the PPHMM instantiated at each branch in the phylogeny is used to re-align the ancestral and descendent taxa at either end of the branch. Because non-leaf taxa are unobservable, this procedure effectively re-aligns species inside the child clade (as a unit) to all species outside the child clade (as a unit). Emission probabilities are computed using a variant of Felsenstein's algorithm which finds the maximum probability over all gain/loss histories; we call this variant *Gain-Loss Felsenstein* (see Text S1). For these down-pass re-alignment steps, a constrained variant of Viterbi decoding is used, which considers only state paths which respect the *functional parse* (assignment of functional classes to residues) of the ancestral sequence; the very first re-alignment step of the down-pass utilizes unconstrained Viterbi decoding to obtain a functional parse for the root sequence (this root decoding step is essentially equivalent to a standard Phylo-HMM). Once the down-pass completes, all sequences (including ancestral sequences) will have been assigned a functional parse, from which binding site predictions are extracted.

Evaluation of emission probabilities in gain and loss states is performed via:
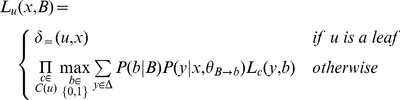
(11)where *P*(*b*|*B*) is given by the birth-death equations described above, *x* is a nucleotide, *C*(*u*) are the children of *u*, Δ is the nucleotide alphabet, and *P*(*y*|*x*,*θ_B_*_→*b*_) imposes the substitution mixture model *θ_B_*_→*b*_ for the current state, *q*. This formula produces a conditional likelihood, *P*(**S** *_leaves_*|*S_root_*,*q*)  =  *L_root_*(*S_root_*,*B*) for *S_root_* the (unobserved) residue at the clade root and **S** *_leaves_* the set of (observed) residues at the leaves of the clade. To obtain a marginal likelihood, *P*(**S** *_leaves_*|*q*), we compute ∑*_x_L_root_*(*x*,*B*)*P_eq_*(*x*|*B*), for *P_eq_*(*x*|*B*) the equilibrium nucleotide frequency obtained from the substitution matrix for the class *B* implied by state *q*.

Detailed descriptions of all algorithms referenced above are given in Text S1.

### Training and Evaluation

For the experiments described here, the following protocols were observed for training and application of the software. Parameters *α* and *β* were trained via simple counts taken from a training alignment. *λ* and *µ* were constrained via *λ* = *δµ*/(1- *δ*) for *δ* the estimated density of binding sites in training data, and then optimized to maximize site-level prediction accuracy on training data (for *Drosophila* experiments *µ* was fixed at 0.08 as early runs indicated little or no advantage to changing this value); because these were trained discriminatively they need not reflect the actual birth and death rates along ancestral lineages. Relative frequencies for individual transcription factors were estimated from training data as well, and were used to scale the probabilities of transitions entering submodels for individual factors; an additional multiplicative term of 0.5 was applied, since we included a forward and reverse-strand submodel for each factor. Two additional multiplicative factors were introduced into the model after it was observed in simulations that they could improve the discriminative power of the predictor: an indel coefficient *c_indel_* which was applied to *α* and *β*, and a branch coefficient *c_branch_*, which was applied to all branch lengths in the phylogeny. The need for such “fudge factors” in improving the discriminative power of generative models has been well-documented, particularly in the context of cross-species gene prediction [9], and more recently in cross-species binding-site prediction [7]. These coefficients were optimized by maximizing the predictive accuracy of the model on the training data via hill-climbing (with site-level *F*-score as the objective function). The background substitution rate matrix was trained via gradient ascent on training alignments; foreground rate matrices (one for each position in each factor's binding profile) were trained via the Halpern-Bruno construction, as mentioned previously. Branch lengths for the phylogeny were estimated simultaneously with the background rate matrix via gradient ascent (since rates and times are confounded). Note that these branch lengths are likely to be less suitable for use in computing the transition and gain/loss probabilities, since they were estimated specifically to maximize the likelihood of the (background) substitution matrix only. This likely accounts for the improvement observed when *c_branch_* was incorporated into the model; in the future we intend to instead estimate separate branch lengths for these other components of the model. The model currently has very few free parameters, despite having many hundreds of states: *α*, *β*, *λ*, *µ*, *δ*, *c_indel_*, *c_branch_*, a relative density for each factor, the phylogeny branch lengths, and the parameters of the background rate matrix (6 for GTR); binding site profiles (positional weight matrices) were obtained from external sources and were not estimated directly by us (though we arbitrarily added pseudocounts of 0.1). Score thresholds for PhyloGibbs and rMONKEY predictions were optimized via cross-validation; overlapping predictions were disambiguated by selecting the highest scoring sites.

For the ROC-like curves, we obtained Bowtie [38] alignments of ChIP-seq reads from Bradley *et al.* [24] and subjected these to the F-Seq program [25]; F-Seq scores were normalized using total-chromatin files provided by Bradley *et al.* [24]. We then applied 1000 cutoff values to the resulting F-Seq profiles to obtain intervals of varying sizes around putative binding sites. At each cutoff we evaluated sensitivity and false positive rates for a fixed set of predictions and plotted these as in a standard ROC curve. The resulting curve was smoothed by averaging values in a fixed-length window; identical smoothing parameters were applied to all curves.

## Supporting Information

Text S1 Detailed description of algorithms and models discussed in the manuscript, additional results, and additional methods.
(1.64 MB DOC)
