# Supplementary material for: Modeling the Evolution of Regulatory Elements by Simultaneous Detection and Alignment with Phylogenetic Pair HMMs
Source: PLoS Comput Biol. 2010 Dec 16;6(12):e1001037. doi: 10.1371/journal.pcbi.1001037 (PMC3002982; doi:10.1371/journal.pcbi.1001037)
Supplement: Text S1 — Detailed description of algorithms and models discussed in the manuscript, additional results, and additional methods. (1.64 MB DOC) [file pcbi.1001037.s001.doc]

**Text S1**

Supplementary Text for:

# Modeling the Evolution of Regulatory Elements by Simultaneous Detection and Alignment with Phylogenetic Pair HMMs

## Majoros WH^1^, Ohler U^1,2^

### ^1^Institute for Genome Sciences & Policy, Duke University, Durham, NC, USA ; ^2^Department of Biostatistics & Bioinformatics, Duke University, Durham, NC, USA

Table of Contents

1. Phylogenetic Pair HMMs [2](#__RefHeading___Toc147383024)

1.1 Basic Definitions [2](#__RefHeading___Toc147383025)

1.2 Aligning Two Sequences [5](#__RefHeading___Toc147383026)

1.3 Aligning Two Alignments [7](#__RefHeading___Toc147383027)

1.4 Hirschberg Decoding Algorithm [9](#__RefHeading___Toc147383028)

2. A Birth-death Process for Binding Sites [12](#__RefHeading___Toc147383029)

3. Variants of Felsenstein Pruning [13](#__RefHeading___Toc147383030)

3.1 Lossy Felsenstein [13](#__RefHeading___Toc147383031)

3.2 Gain-Loss Felsenstein [14](#__RefHeading___Toc147383032)

4. Modeling Language [14](#__RefHeading___Toc147383033)

5. Additional Results [16](#__RefHeading___Toc147383034)

5.1 EVOS Simulations [16](#__RefHeading___Toc147383035)

5.2 One Hundred Forty Two RedFly CRMs [19](#__RefHeading___Toc147383036)

5.3 Seventeen RedFly CRMs [20](#__RefHeading___Toc147383037)

6. Binding-site Profiles [22](#__RefHeading___Toc147383038)

6.1 Weight Matrices from JASPAR [22](#__RefHeading___Toc147383039)

6.2 Weight Matrices from EMMA Package [23](#__RefHeading___Toc147383040)

References [25](#__RefHeading___Toc147383041)

# 1. Phylogenetic Pair HMMs

## **1.1 Basic Definitions**

A *Pair HMM* for DNA sequences is defined as a special type of HMM denoted by a 7-tuple:

| M=(*q*^0^,Q_M_,Q_I_,Q_D_,*α*,P_t_,P_e_), | (1.1) |
| --- | --- |

for state set Q=Q_M_∪Q_I_∪Q_D_∪{q^0^}, DNA alphabet *α*, transition distribution P_t_:Q×Qa¡, silent initial/final state q^0^, and emission distribution P_e_:*α*_+_×*α*_+_×Qa¡, for the augmented alphabet *α*_+_={A,C,G,T,-}. Given a numbering of the states in *Q*, {*q*_0_,...,*q_n_*}, we assume for convenience that *q*_0_=*q*^0^. All emission probabilities in *q*_0_ are 0:

| *P_e_*(*s*∈*α*_+_,*t*∈*α*_+_|*q*_0_) = 0, | (1.2) |
| --- | --- |

All the state sets Q_M_, Q_I_, Q_D_, and {q_0_} are disjoint. States in Q_M_ are referred to as *match states*, and are subject to:

| *P_e_*(*s*∈*α*_+_,-|*q*∈*Q*_M_) = *P_e_*(-,*s*∈*α*_+_|*q*∈*Q*_M_) = 0, | (1.3) |
| --- | --- |

though it should be clear that these so-called “match” states can emit non-identical pairs of symbols as well as identical pairs. States in Q_I_ are known as *insertion states*, and satisfy:

| *P_e_*(*s*∈*α*,*s*∈*α*_+_|*q*∈*Q*_I_) = *P_e_*(-,-|*q*∈*Q*_I_) = 0, | (1.4) |
| --- | --- |

while states in Q_D_ are known as *deletion states*, and satisfy

| *P_e_*(*s*∈*α*_+_,*s*∈*α*|*q*∈*Q*_D_) = *P_e_*(-,-|*q*∈*Q*_D_) = 0, | (1.5) |
| --- | --- |

so that insertion states can emit only pairs from {-}×*α*, while the deletion states emit only pairs from *α*×{-}. We forbid the emission of (-,-) from any state, as in traditional sequence alignment:

| *P_e_*(-,-|*q*∈*Q*)=0. | (1.6) |
| --- | --- |

It is also sometimes desirable to forbid transitions between insertion and deletion states:

| *P_t_*(*q_i_*∈*Q_I_*|*q_j_*∈*Q_D_*) = *P_t_*(*q_j_*∈*Q_D_*|*q_i_*∈*Q_I_*) = 0. | (1.7) |
| --- | --- |

though this is not necessary. We will denote the emitting states as *Q_E_*=*Q*–{*q*^0^}.

## **1.2 Algorithms Relating Parses and Alignments**

The objective function for MAP (*maximum a posteriori*) decoding can be stated as:

|  | (1.8) |
| --- | --- |

for *y_i_*∈*Q*, *y*_0_= *y_m_*_-1_=*q*_0_, where *a_i_*,*_j_* is the *i*^th^ symbol in the *j*^th^ sequence (*j*∈{1,2}) of the resulting alignment *A*(*φ*). These *a_i_*,*_j_* are unambiguously defined for any given parse *φ*={*y*_0_,...,*y_m_*_-1_}, as given by Algorithm 1.1.

**Algorithm 1.1** Reconstructing an alignment *A*={*a_i_*,*_j_*} from a PairHMM parse, *φ*. *S*_1_ and *S*_2_ are the sequences being aligned; Q_I_, Q_D_, and Q_M_ are the insertion, deletion, and match states, respectively, of the PairHMM.

**procedure** alignmentFromParse(*φ*,Q_M_,Q_I_,Q_D_,*S*_1_,*S*_2_)

1. (k_1_,k_2_)←(0,0);
2. **for** i←1 **up** **to** |*φ*| **do**
3. **if** *φ*[i]∈Q_I_ **then**
4. (*a_i_*_,1_,*a_i_*_,2_)←(“-”,S_2_[k_2_]);
5. k_2_←k_2_+1;
6. **else if** *φ*[i]∈Q_D_ **then**
7. (*a_i_*_,1_,*a_i_*_,2_)←(S_1_[k_1_],“-”);
8. k_1_←k_1_+1;
9. **else**
10. (*a_i_*_,1_,*a_i_*_,2_)←(S_1_[k_1_],S_2_[k_2_]);
11. (k_1_,k_2_)←(k_1_+1,k_2_+1);
12. **return** *a*;

The inverse problem is solved by Algorithm 1.2, in the special case of a 4-state PairHMM in which *Q_I_*={*q_I_*}, *Q_D_*={*q_D_*}, *Q_M_*={*q_M_*}.

**Algorithm 1.2** Reconstructing a PairHMM parse *φ* from an alignment *A*={*a_i_*,*_j_*} of length *L*, in the special case: *Q_I_*={*q_I_*}, *Q_D_*={*q_D_*}, *Q_M_*={*q_M_*}.

**procedure** parseFromAlignment3(*A,L*)

1. *φ*←(*q*_0_);
2. **for** i←0 **up** **to** *L*-1 **do**
3. **if** *A*[i][1]=“-” **then push** *φ*,*q_I_*;
4. **else** **if** *A*[i][2]=“-” **then push** *φ*,*q_D_*;
5. **else** **push** *φ*,*q_M_*;
6. **push** *φ*,*q*_0_;
7. **return** *φ*;

This algorithm is applicable only to 3-state PHMMs having a single insertion, deletion, and match state (each). When *Q_I_*, *Q_D_*, or *Q_M_* contains more than one state, additional information is needed to disambiguate state identities, as we describe shortly.

We first extend the PairHMM formulation to account for functional classes of states. In our model of transcription factor binding sites, each position of each transcription factor profile is represented by a different functional class; there is also a background functional class denoting the absence of any binding site.

Let us denote a *PairHMM with functional classes* as a 9-tuple:

| M=(*q*^0^,Q_M_,Q_I_,Q_D_,*α*,P_t_,P_e_,*C,f_C_*), | (1.9) |
| --- | --- |

in which all components are defined as for Eq. (1.1), except that *C*={*c*_0_,*c*_1_,...} is a set of *functional classes*, and *f_C_*:*Q*a*C* assigns a unique class to each state. We also require that each functional class have at most one insertion, deletion, and match state (each) associated with it:

|  | (1.10) |
| --- | --- |

for the inverse relation $f_{C}^{-1}(c)$={*q*|*f_C_*(*q*)=*c*}—i.e., the set of all pre-images of *c*.

To avoid confusion, let us refer to any parse (i.e., state sequence) from a PairHMM as a *state parse*; we can denote a state parse with the symbol *φ*. We differentiate between this latter construct and that of a *functional parse*, by considering a functional parse to be a sequence of functional classes Φ=(*v*_0_,...,*v_m_*_-1_), *v_i_*∈*C* for all 0≤*i*<*m*, rather than a sequence of states as in a state parse. A functional parse may correspond to a particular sequence or to an alignment of two or more sequences; in the latter case it is understood that each of the individual sequences has a functional parse associated with it, as implied by the functional parse for the alignment in which that sequence participates.

Given a state parse *φ*={*y*_0_,...,*y_m_*_-1_}, we can obtain the corresponding functional parse Φ via:

| Φ={*f_C_*(*y*_0_),*f_C_*(*y*_1_),...,*f_C_*(*y_m_*_-1_)}. | (1.11) |
| --- | --- |

Now we can reconstruct a state parse from an alignment in the general case (subject to the constraints in Eq. 1.10), if we are also given a functional parse Φ, using Algorithm 1.3.

**Algorithm 1.3** Reconstructing a state parse *φ* from an alignment *A*={*a_i_*,*_j_*} of length *L*, given a functional parse Φ also of length *L*. Procedure elem(s) returns the sole element of a singleton set s.

**procedure** stateParseFromAlignment(*A,L,*Φ)

1. *φ*←(*q*_0_);
2. **for** i←0 **up** **to** *L*-1 **do**
3. s←f_C_^-1^(Φ[i]);
4. **if** *a*[i][1]=“-” **then push** *φ*,elem(s∩*Q_I_*);
5. **else** **if** *a*[i][2]=“-” **then push** *φ*,elem(s∩*Q_D_*);
6. **else** **push** *φ*,elem(s∩*Q_M_*);
7. **push** *φ*,*q*_0_;
8. **return** *φ*;

Extracting the functional parse Φ*_j_* for just one of the two sequences *S_j_* involved in a pairwise alignment *A*={*a_i_*_,1_,*a_i_*_,2_|0≤*i*<*m*} is as simple as removing from Φ any element *c_i_* for which *a_i,j_* is a gap. The inverse operation can also be performed—i.e., associating a class *c_i_* from position *i* of sequence *S_j_* with the corresponding position in a pairwise alignment involving *S_j_*—though alignment positions associated with a gap in *S_j_* will not receive any class this way. Given functional parses Φ_1_ and Φ_2_ for parent and child sequence *S*_1_ and *S*_2_, and a pairwise alignment *A*, we can use this procedure to deduce a functional parse Φ for *A* by taking classes from Φ_1_ and Φ_2_ for the non-gap positions of *S*_1_ or *S*_2_, respectively; obviously, if Φ_1_ and Φ_2_ are not consistent, then Φ will not be unambiguously defined.

Let *f_A_^x^_S_*(*i*,*A*) denote a mapping from positions in a pairwise alignment *A* to corresponding positions in sequence *S_x_*, *x*∈{0,1}, and let *f_S_^x^_A_*(*i*,*A*) denote the mapping from positions in sequence *S_x_* to corresponding positions in *A*; we may substitute a state parse *φ* for *A* in either of these terms, since Algorithm 1.1 provides a way of obtaining *A* from *φ*. Algorithm 1.4 computes *f_A_^x^_S_*(*i*,*A*) and *f_S_^x^_A_*(*i*,*A*) from a given pairwise alignment *A*.

**Algorithm 1.4** Mapping positions to or from an alignment *A*={*a_i_*,*_j_*} of length *L*.

**procedure** getPositionMaps(*A,L*)

1. i←j←-1;
2. **for** k←0 **up** **to** *L*-1 **do**
3. **if** *a*[i][1]=“-” **then** j←j+1;
4. **else** **if** *a*[i][2]=“-” **then** i←i+1;
5. **else** (i,j)←(i+1,j+1);
6. f_S_^1^_A_(i,A)←f_S_^2^_A_(j,A)←k;
7. f_A_^1^_S_(k,A)←i;
8. f_A_^2^_S_(k,A)←j;
9. **return** (f_S_^1^_A_,f_S_^2^_A_,f_A_^1^_S_,f_A_^2^_S_);

Now functional parse Φ*_j_* for sequence *S_j_* involved in pairwise alignment *A* (having functional parse Φ) can be extracted via:

|  | (1.12) |
| --- | --- |

The inverse problem is solved via:

|  | (1.13) |
| --- | --- |

In order to allow for evolutionary gain and loss of binding sites, we introduce the notion of a *cross-functional state*, or CFS, in which the functional class of the residue in the parent sequence may differ from that of the orthologous residue in the child taxon. The existence of cross-functional states only slightly complicates the above formulation of the PairHMM. Each state will now have two functional classes associated with it: the *parent class* and the *child class*, denoted *f_C_^parent^*(*q*) and *f_C_^child^*(*q*), respectively; alternatively, we can take *f_C_*(*q*) to evaluate to a pair:

| *f_C_*(*q*) = (*f_C_^parent^*(*q*),*f_C_^child^*(*q*)). | (1.14) |
| --- | --- |

Algorithm 1.3 as given above remains unaffected by the existence of cross-functional states if we treat elements of Φ as pairs; changes to other algorithms to accommodate CFS will be detailed where necessary.

## **1.3 Aligning Two Sequences**

An optimal pairwise alignment between two sequences can be computed by performing MAP decoding of a PairHMM:

|  | (1.15) |
| --- | --- |

A dynamic programming recursion for MAP decoding can be devised by observing the following discipline. We utilize a DP matrix *V_i_*,*_j_*,*_k_*, where the first two subscripts correspond to positions in the two sequences, and the third subscript specifies a state. *V_i_*,*_j_*,*_k_* denotes the hypothesis that the machine will reside in state *q_k_* at precisely the point in time when *s*_0,1_...*s_i_*_-1,1_ and *s*_0,2_...*s_j_*_-1,2_ have been emitted. A valid parse must start in cell *V*_0,0,0_, since the machine always begins in state *q*_0_. For any cell *V_i_*,*_j_*,*_k_*, the optimal predecessor will be one of *V_i_*,*_j_*_-1_,*_h_* (for insertions), *V_i_*_-1_,*_j_*,*_h_* (for deletions), or *V_i_*_-1_,*_j_*_-1_,*_h_* (for matches or mismatches), with the optimal predecessor state *q_h_* chosen so as to maximize these terms (weighted by the probability of state transition *q_h_*→*q_k_* and the corresponding emission upon reaching *q_k_*), subject to the constraint that insertions, deletions, and matches/mismatches occur only when the machine transitions into a state in *Q_I_*, *Q_D_*, or *Q_M_*, respectively. We thus arrive at the following recursion formulae:

|  | (1.16) |
| --- | --- |

for 0<*i*≤|*S*_1_|, 0<*j*≤|*S*_2_|, and 0≤*k*<|*Q*|, where the following initializations are assumed:

|  | (1.17) |
| --- | --- |

for all 0≤*k*<|*Q*|, where *V* is a three-dimensional real matrix of size (*m*+1)×(*n*+1)×|*Q*|, for *m*=|*S*_1_| and *n*=|*S*_2_|, and *s_i_*,*_j_* is the *i*^th^ symbol in the *j*^th^ input sequence—these are distinct from the *a_i_*,*_j_* described above, as the index *i* is relative to the beginning of the raw sequence, not the current partial alignment. Note that the matrix is wider and deeper than the respective lengths of the two sequences, since indexes of zero in the first two dimensions do not correspond to any positions in the emitted sequences. Thus, each cell *V_i_*,*_j_*,*_k_* contains the joint probability of the sequences *s*_0,1_... *s_i_*_-1,1_ and *s*_0,2_...*s_j_*_-1,2_ and the most probable path ending in state *q_k_* that would emit precisely these sequences.

The matrix *T*, of the same dimensions as *V*, stores *traceback pointers* (integer state indices) to facilitate reconstruction of the optimal parse, as in traditional Viterbi decoding:

|  |  |
| --- | --- |

Given *φ^*^*, the denoted alignment can be explicitly constructed via Algorithm 1.1 from the previous section.

## **1.4 Aligning Two Alignments**

Aligning two alignments (or “profiles”), *A_Y_* and *A_Z_*, to each other to produce a composite alignment *A_X_* can be accomplished via the MAP decoding procedure for PairHMMs given above, if we redefine *P_e_*(*s*_1_,*s*_2_|*q*) so as to utilize Felsenstein’s algorithm to evaluate the probability of emitting an entire column of residues rather than just a pair—i.e., replacing *P_e_*(*s*_1_,*s*_2_|*q*) with *P_e_*(**s***_Y_*,**s***_Z_*|*q*), for **s***_Y_* and **s***_Z_* vectors assigning residues (or gap symbols) to particular nodes within clades T*_Y_* and T*_Z_*, respectively.

Formally, suppose we have a non-leaf node *X* having children *Y* and *Z*, and suppose that the alignments corresponding to the clades rooted at *Y* and *Z* are, respectively, denoted *A_Y_* and *A_Z_*; if either of *Y* or *Z* are leaves, their corresponding alignments will consist of just the sequence of that leaf. Let T*_X_* denote the phylogeny for the clade rooted at *X* (and similarly for T*_Y_* and T*_Z_*), the leaves of which are assumed to be ordered so they may be placed in one-to-one correspondence with elements of a vector of appropriate size. We denote by T*_Y_*B**s***_Y_* the assignment of residues from vector **s***_Y_* to their corresponding variables (i.e., nodes) in T*_Y_*. Given T*_Y_*B**s***_Y_* and T*_Z_*B**s***_Z_* for a particular pair of columns *i* and *j* in alignments *A_Y_* and *A_Z_*, respectively, the term *P_e_*(*Y*,*Z*|*q*) = *P_e_*(**s***_Y_*,**s***_Z_*|*q*) can be evaluated by applying Felsenstein’s algorithm to T*_X_*:

|  | (1.18) |
| --- | --- |

where *P_eq_*(*X*=*x*) is the equilibrium frequency of residue *x*, and the substitution probabilities used within *L_X_*() are specified by the evolution model *_q_* associated with state *q*. Generalization of the terms *P_e_*(*s*_1_,-|*q*) and *P_e_*(-,*s*_2_|*q*) can be obtained similarly by setting all elements of **s***_Z_* or **s***_Y_*, respectively, to the gap symbol.

Recall that Felsenstein’s algorithm computes the following recurrence:

|  | (1.19) |
| --- | --- |

where *δ*= is the *Kronecker delta function*, which evaluates to 1 if the parameters are equal and 0 if they are not equal, *C*(*u*) is the set of children of node *u*, and Δ is the DNA alphabet. An augmented version of Felsenstein’s recurrence which accommodates cross-functional states for TFBS loss and/or gain is given by:

|  | (1.20) |
| --- | --- |

where *θ_B_*_→_*_b_* is a *mixture substitution model* (see below), and *P*(*b*|*B*) is given by:

|  | (1.21) |
| --- | --- |

where columns specify the child class (*b*), rows specify the parent class (*B*), *α* is given by:

|  | (1.22) |
| --- | --- |

and *β* is given by:

|  | (1.23) |
| --- | --- |

Parameters *λ* and *μ* are the birth and death rates for TFBS elements (*not* individual nucleotides), to be described in section 1.4. The binary indicators *B* and *b* (with 0 representing TFBS absent and 1 representing TFBS present) can be generalized to arbitrary functional classes, though additional parameters (and the solutions to more differential equations) are then required in order to model the transition rates between functional classes.

The termination formula for the recurrence in Eq. (1.20) is:

|  | (1.24) |
| --- | --- |

for root residue *x* and root binary presence indicator *B*; in the case of an unobservable root, summation over *x* and *B* should be performed.

The mixture substitution model *θ_B_*_→_*_b_* is formally defined as:

|  | (1.25) |
| --- | --- |

where **Q***_B_* and **Q***_b_* are the instantaneous rate matrices for the parental class *B* and child class *b*, respectively, and *t* is the branch length for the branch connecting the two taxa in the phylogeny. Eq. (1.25) accounts for the unknown time *s* at which the lineage changes class from *B* to *b*, with the substitution rates undergoing a simultaneous change at that time.

A solution to Eq. (1.25) can be derived as follows. Let the spectral decomposition of the respective substitution matrices be given as **Q***_B_*=**M**_1_**Λ**_1_**M**_1_^-1^ and **Q***_b_*=**M**_2_**Λ**_2_**M**_2_^-1^, for **M***_i_* a matrix of column eigenvectors and **Λ***_i_* a diagonal matrix of corresponding eigenvalues. Then we have:

|  | (1.26) |
| --- | --- |

for **M**_3_=**M**_1_^-1^**M**_2_=[*m_ij_*], **Λ***_k_*=diag(*λ_k_*_1_,*λ_k_*_2_,*λ_k_*_3_,*λ_k_*_4_), and where *B_ij_* is given by:

|  | (1.27) |
| --- | --- |

since:

| . | (1.28) |
| --- | --- |

## **1.5 Hirschberg Decoding Algorithm**

The Hirschberg algorithm allows computation of the Viterbi path in limited memory with only a constant factor penalty in time complexity. We begin with the *forward Hirschberg recurrence*, which is essentially the same as the Viterbi recurrence. The global initialization of the forward recurrence is given by:

|  | (1.29) |
| --- | --- |

The forward Hirschberg procedure is applied recursively to intervals *s_a_*_,1_...*s_b_*_,1_ from the first input sequence (*S*_1_) and *s_c_*_,2_...*s_d_*_,2_ from the second input sequence (*S*_2_). As a precondition, *V_a,c,k_* is assumed to be precomputed for all states *q_k_*; for the first iteration of Hirschberg, this precondition is met via (Eq. 1.29), while for later iterations *V_a,c,k_* will have been computed (and preserved) during a previous iteration.

The procedure begins with two initialization steps. First, from *V_a,c,k_* we compute the rest of column *c*:

|  | (1.30) |
| --- | --- |

The second initialization step is then applied just prior to each subsequent application of the recurrence (in column *j*, for *c*<*j*≤*d*), to compute *V_a_*,*_j_*,*_k_* for all states *q_k_*:

|  | (1.31) |
| --- | --- |

The actual recurrence is:

|  | (1.32) |
| --- | --- |

for *a*<*i*≤*b*, *c*<*j*≤*d*, and 0≤*k*<|*Q*|; during the first iteration of Hirschberg we would obviously have *a*=0, *b*=*m*, *c*=0, and *d*=*n*, where *m* and *n* are the lengths of the input sequences (*m*=|*S*_1_|, *n*=|*S*_2_|).

The global initialization of the *reverse Hirschberg recurrence* is given by:

|  | (1.33) |
| --- | --- |

Each iteration imposes the precondition that *R_b,d,k_* has been precomputed for all states *q_k_*; for the first iteration of Hirschberg, this precondition is met via (Eq. 1.33), while for later iterations *R_b,d,k_* will have been computed (and preserved) during a previous iteration.

The procedure begins with two initialization steps. First, from *R_b,d,k_* we compute the rest of column *d*:

|  | (1.34) |
| --- | --- |

The second initialization step is then applied just prior to each subsequent application of the recurrence (in column *j*, for *c*≤*j*<*d* ), to compute *R_b,j,k_* for all states *q_k_*:

|  | (1.35) |
| --- | --- |

The actual recurrence is:

|  | (1.36) |
| --- | --- |

for *a*≤*i*<*b*, *c*≤*j*<*d*.

As a result of these forward and reverse recurrences, we have:

|  | (1.37) |
| --- | --- |

so that:

|  | (1.38) |
| --- | --- |

where *P_max_* denotes the *maximal probability* (i.e., for the *optimal path*), and *χ_i,j,k_* indicates that cell (*i*,*j*,*k*) lies on that optimal path.

*V_i,j,k_R_i,j,k_* thus gives the maximal path score for paths passing through (*i*,*j*,*k*). Choosing the maximum such score for a given column *j^*^* will give the *globally* maximal path score, and identifies the cell through which that path must pass. Hirschberg recursively partitions the interval (0,*n*) into sub-intervals by computing the *V_i,j*,k_* and *R_i,j*,k_* values for the midpoint *j^*^*, as follows.

Given that the current problem is to decode over intervals (*a*,*b*) in the first sequence and (*c*,*d*) in the second sequence, we pick a *split column*, *j^*^*, via:

|  | (1.39) |
| --- | --- |

Initially, *a*=0, *b*=*m*, *c*=0, and *d*=*n*. The optimal *crossing point* (row *i* and state *q_k_*) for column *j^*^* is specified by:

|  | (1.40) |
| --- | --- |

Once the optimal crossing point has been identified, all competing crossing points must be zeroed out—i.e., all entries in *V* and *R* other than *V_i*,j*,k*_* and *R_i*,j*,k*_* (from this or any previous iteration).

The problem can now be subdivided into a *left subproblem*:

|  | (1.41) |
| --- | --- |

and a *right subproblem*:

|  | (1.42) |
| --- | --- |

each of which give new (*i^*^*,*k^*^*) pairs to associate with the new split columns. These triples (*i^*^*,*j^*^*,*k^*^*) constitute the optimal Viterbi path. Enumeration of all the triples in the optimal path can be accomplished via recursion to the left and right subproblems. This recursion ends when *c*=*d* or *c*=*d*-1. In both cases, the forward Viterbi matrix consists of no more than two columns, so we can simply apply the forward Viterbi recurrence and then backtrack through the one or two columns still populated after application of the recurrence. Implementation of this procedure in conjunction with banding of the dynamic programming matrix requires additional care to avoid indexing into undefined cells in the matrix.

# 2. A Birth-death Process for Binding Sites

We now consider a birth-death process on TFBSs. In this model, a TFBS observed to exist in an ancestral taxon will, over an interval of *t* time units, either survive to the end of the interval (and possibly beyond) or die before the end of the interval. We define the following variables:

- *p*(*t*) = the probability that the TFBS is present at the end of the interval
- *q*(*t*) = the probability that the TFBS is *not* present at the end of the interval

In addition, for any vacant site in the ancestor (i.e., having no binding functionality), we define:

- *b*(*t*) = the probability that a TFBS is present at this site at the end of the interval

From these we derive the following system of differential equations:

|  | (2.1) |
| --- | --- |

|  | (2.2) |
| --- | --- |

|  | (2.3) |
| --- | --- |

for *μ* the instantaneous death rate and *λ* the instantaneous birth rate for whole binding sites. Boundary conditions for the above system are *p*(0)=1, *q*(0)=0, *b*(0)=0, and *p*(*t*)+*q*(*t*)=1.

A solution to the above equations is given by:

|  | (2.4) |
| --- | --- |

|  | (2.5) |
| --- | --- |

|  | (2.6) |
| --- | --- |

For the experiments described in the main article, we constructed a PPHMM having a gain and loss submodel for each transcription factor included in the model. The topologies of the gain and loss models are shown in Figure 2 in the main article. Variables *a* through *r* in that figure denote transition probabilities taken from the background model, with the precise correspondence as depicted below:


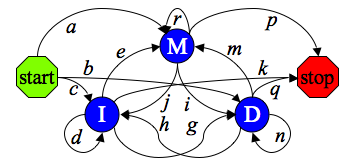


**Figure S1** : Generic background PPHMM. Variables labeling transitions indicate the correspondence between transitions in Figures 1 and 2 in the main article.

# 3. Variants of Felsenstein Pruning

## **3.1 Lossy Felsenstein**

During the up-pass of progressive alignment, emission terms for Viterbi/Hirschberg are computed using a “lossy Felsenstein” algorithm, F*_L_* (and then cached for efficiency). This algorithm permits retention or loss of function, but not gain, and may improve the ability for evidence of binding sites low in the phylogeny to continue being propagated higher up the tree, where they may meet similar evidence from other branches.

The recursion for F*_L_* is:

|  | (3.1) |
| --- | --- |

where *F* is the foreground functional class under consideration by Viterbi/Hirschberg for some taxon, and *B* is the background functional class (only one such background class is assumed to exist; many foreground classes will generally exist, but a single state under consideration in Viterbi/Hirschberg dictates a single foreground class for each of the two taxa being aligned), and Φ*_u_*[*i*]=*Z* evaluates to true if the functional class assigned to the *i*^th^ residue in taxon *u*’s sequence is *Z*, or false if either the assigned class is not *Z* or if no class has been assigned (meaning that the functional parse of *u* is as yet unresolved). *P*(*B*|*B*) and *P*(*Z*|*F*) were omitted in the computational experiments because early simulation results suggested better discriminative power without these terms.

Whereas the traditional Felsenstein algorithm requires a matrix of size |*T*|×|Δ|, for taxa *T* and alphabet Δ, the lossy version requires a three-dimensional dynamic programming matrix of size |*T*|×|Δ|×2. The run time and space requirements are thus only greater than traditional Felsenstein by a constant factor. For the more general algorithm which permits gains and losses of all classes, the matrix size would be |*T*|×|Δ|×|*C*| for *C* the set of all functional classes; for models containing all known mammalian transcription factor motifs (for example), *C* will be roughly 80*L_ave_*, for *L_ave_* the average motif length. Thus, the more general model can be substantially more computationally expensive than F*_L_*.

## **3.2 Gain-Loss Felsenstein**

During both the down-pass of progressive alignment and during the optional refinement phase, emission terms for Viterbi are computed using a variant of Felsenstein that we call *Gain-Loss Felsenstein*, or F*_GL_*, which permits both gains and losses of function along a lineage, but only for a single functional class. That is, F*_GL_* permits any number of switches between the background functional class *B* and a single foreground functional class *F*.

The recursion for F*_GL_* is:

|  | (3.2) |
| --- | --- |

Whereas the up-pass left all functional parses unresolved so as to avoid greedy behavior, the purpose of the down-pass is to resolve the functional parses of all taxa once information has been globally propagated to the root (by the up-pass). Thus, as the down-pass progresses, some of the functional parses will have been resolved and others will not. F*_GL_* thus needs to respect the functional classes that have already been assigned to residues, while (ideally) considering all possible functional classes for residues not yet assigned a functional class. As noted above, this would require a matrix of size |*T*|×|Δ|×|*C*| for taxa *T*, alphabet Δ, and the set of all functional classes (*C*). To mitigate memory requirements in the case of large |*C*|, we instead consider in F*_GL_* all possible assignments of classes from the set {*F*,*B*}, for *B* the background class and *F* a single foreground class currently under consideration by Viterbi. F*_GL_* thus evaluates to the maximum likelihood for the ensemble of all unresolved residues within the current column, allowing for any number of gain and loss events along a lineage for a given foreground class.

# 4. Modeling Language

Our system implements a novel programming language, *SEAL* (*Simple Evolutionary Alignment Language*), which can be used to specify both evolutionary dynamics and probabilistic alignment algorithms. Like LISP and Scheme, SEAL is based on the *lambda calculus* (Church, 1932). It has only four grammatical constructs: [x y z| ... ] defines a function closure taking three parameters, (func a b c) is a function call, ’abc is a symbol, and abc is a variable. MAFIA’s SEAL implementation pre-defines primitives for specifying PPHMM templates and registering them with MAFIA. Given a phylogeny Ψ, MAFIA instantiates the given template on each branch, resulting in a PPHMM with transition and emission parameters scaled according the branch on which it resides. Transition probabilities are defined by the user as function closures parameterized by branch length: (transition X Y [t|...]), where the “...” can be an arbitrary mathematical formula. In this way, indel models such as TKF can be specified compactly. Example code implementing our reversible indel model is shown below:

(define 'eT [x t|(exp (- 0 (* x t)))])

(define 'emT [x t|(- 1 (eT x t))])

(define 'bINF [lambda mu|(/ (float lambda) (+ lambda mu))])

(define 'bT [t lambda mu|(*(bINF lambda mu)(emT (+ lambda mu) t))])

(define 'stop [t lambda mu|(- 1 (*(- 1 (bINF lambda mu))(- 1 (bT t lambda mu))))])

(define 'nonstop [t lambda mu x|(* x (- 1 (stop t lambda mu)))])

(define 'reversible [alpha beta L mu fc|([M I D|(transducer

(states M I D)

(transition start-state I [t|(/ (emT alpha t) 2)])

(transition start-state D [t|(/ (emT alpha t) 2)])

(transition start-state M [t|(eT alpha t)])

(transition M M [t|(nonstop t L mu (eT alpha t))])

(transition M I [t|(nonstop t L mu (/ (emT alpha t) 2))])

(transition M D [t|(nonstop t L mu (/ (emT alpha t) 2))])

(transition M end-state [t|(stop t L mu)])

(transition I I [t|(nonstop t L mu (emT beta t))])

(transition I M [t|(nonstop t L mu (* (eT beta t) (eT alpha t)))])

(transition I D [t|(nonstop t L mu (* (eT beta t) (emT alpha t)))])

(transition I end-state [t|(stop t L mu)])

(transition D D [t|(nonstop t L mu (emT beta t))])

(transition D M [t|(nonstop t L mu (* (eT beta t) (eT alpha t)))])

(transition D I [t|(nonstop t L mu (* (eT beta t) (emT alpha t)))])

(transition D end-state [t|(stop t L mu)]))]

(state 1 MATCH fc)(state 2 INSERT fc)(state 3 DELETE fc))])

A *metamodel* facility (e.g., Majoros, 2007: section 6.6.2) permits the definition of submodels which can then be merged automatically, simplifying the design of complex models having many states. The sample code below illustrates the use of macrostate merging, the definition of functional classes, and the registration of the PPHMM template with the system for a simple two-factor, single-strand model:

(define 'fet [a b c d e|(functional-element-type a b (load-rate-matrices c e) d)])

(load "parms.lambda")(load "reversible.lambda")

(define 'phy (load-phylogeny "drosophila.phy"))

(define 'bg (background (load-rate-matrix "bg.matrix" background-factor) phy "." "bg"))

(define 'fBCD (fet "bcd" "B" "bcd.ratmat" phy site-factor))

(define 'fCAD (fet "cad" "C" "cad.ratmat" phy site-factor))

(define 'pT [t lambda mu|(*(bINF lambda mu)(+ 1 (*(/ mu lambda)(eT (+ mu lambda) t))))])

(define 'qT [t lambda mu|(- 1 (pT t lambda mu))])

(define 'retainP [t lambda mu ratio|(/(*ratio(*(*(- 1 epsilon)(bINF lambda mu))

(pT t lambda mu)))(* 2 (stop t lambda mu)))])

(define 'gainP[t lambda mu ratio|(/(* ratio (*(*(- 1 epsilon)(- 1 (bINF lambda mu)))

(bT t lambda mu)))(* 2 (stop t lambda mu)))])

(define 'lossP [t lambda mu ratio|(/(* ratio (*(*(- 1 epsilon)(bINF lambda mu))

(qT t lambda mu)))(* 2 (stop t lambda mu)))])

(define 'parser[|([spacer|([SPACER BCD CAD |(compose

(macrostates SPACER BCD CAD)

(macrotrans start SPACER [t|1])

(macrotrans SPACER end [t|epsilon])

(macrotrans SPACER BCD [t|(retainP t lambda mu ratio-bcd)])

(macrotrans SPACER CAD [t|(retainP t lambda mu ratio-cad)])

(macrotrans BCD SPACER [t|1])

(macrotrans CAD SPACER [t|1]))

(macrostate 1 spacer)

(macrostate 2 (binding-site fBCD))

(macrostate 3 (binding-site fCAD)))]

(reversible alpha beta lambda mu bg))])

(register-constructor parser)

# 5. Additional Results

## **5.1 EVOS Simulations**

In consideration of the relatively inflexible nature of PSPE for modeling of binding site gains and losses, we also tested MAFIA on sequences produced via another simulator. This simulator, EVOS, utilizes the same generative formalism as MAFIA (a PPHMM), thereby allowing simulation of arbitrary numbers of binding sites undergoing any number of gain and loss events. Because EVOS and MAFIA share much of their underlying software base, we did not test competing software systems on these simulation runs, in the interest of fairness.

For these simulations we utilized a 10-species Drosophila phylogeny: ((((((*dm3*, *sim1*), (*yak2*, *ere2*)), *ana3*), *dp4*), *wil1*), ((*moj3*, *vir3*), *gri2*)); sequence models in the simulator were trained from real Drosophila CRMs and factor weight matrices (factors: *bicoid*, *caudal*, *giant*, *hunchback*, *knirps*, *kruppel*, *tailless*) obtained from a previous study by He *et al.* (2009). Two sets of simulations were produced. In the first set, EVOS applied a scaling factor in the range [0.01,90] to the branch lengths in the phylogeny, which in turn affected indel rates, substitution rates, and binding-site turnover rates; scaling factors were chosen at regular intervals in the above range, producing 108 simulated CRMs. For the second set of simulated CRMs, *λ* and *μ* (binding site *birth* and *death* rates) were varied so as to effect different binding-site densities and degrees of orthology (see below); *λ* was varied in ten increments from 0.001 to 0.01, while *μ* was varied in ten increments from 0.01 to 0.1; all combinations were formed, for a total of 100 simulations. These two data sets differed from each other substantially in several key measures, including *degree of orthology* (percentage of leaf taxa retaining each binding site) and *gappiness* (percentage of alignment positions that are gaps instead of residues), with the first data set having much higher average gappiness (36% versus 11%) and the second set having higher average degree of orthology (66% versus 52%).

Detailed statistics for the EVOS simulation runs are given in Table S1. The table provides measures of *degree of orthology*, *gappiness*, and *conservation* ((*H_max_*-*H*)/*H_max_*, for nucleotide entropy *H*, averaged over alignment columns; *H_max_*=log_2_4). *Foreground conservation* includes all columns overlapping any binding site; *background conservation* includes only columns not overlapping any binding site.

|  | **data set #1** | | | **data set #2** | | |
| --- | --- | --- | --- | --- | --- | --- |
|  | **mean** | **SD** | **range** | **mean** | **SD** | **range** |
| gappiness | 0.36 | 0.36 | 0 – 0.93 | 0.11 | 0.04 | 0.04 – 0.19 |
| average degree of orthology | 0.52 | 0.39 | 0.06 – 1.0 | 0.66 | 0.14 | 0.05 – 0.94 |
| sites/kb | 8.2 | 4.2 | 1.1 – 20.0 | 42.9 | 19.6 | 0.11 – 85.7 |
| overall conservation | 0.85 | 0.12 | 0.63 – 1.0 | 0.80 | 0.02 | 0.75 – 0.84 |
| foreground conservation | 0.79 | 0.20 | 0.38 – 1.0 | 0.83 | 0.02 | 0.77 – 0.89 |
| background conservation | 0.86 | 0.11 | 0.65 – 1.0 | 0.76 | 0.02 | 0.72 – 0.81 |

**Table S1** : Alignment statistics for two sets of EVOS simulations. Gappiness: percentage of alignment positions that are gaps. Average degree of orthology: percentage of leaf tracks in alignment that retain each functional binding site, averaged over all binding sites. Conservation: average of (max entropy – entropy) / (max entropy) over all columns of alignment. Foreground: alignment columns overlapping a binding site. Background: alignment columns not overlapping any binding site.

Prior to running MAFIA, we pre-scanned all input sequences with a weight matrix for each factor, with a relatively liberal threshold (as high as 5% false negative rate), and used these predictions to constrain the Viterbi/Hirschberg dynamic programming matrix so as to reduce the number of states which needed to be considered for each position in each leaf sequence. This reduced the run-time of the system without substantially reducing the predictive accuracy on preliminary simulation studies (data not shown).

MAFIA was parameterized identically to EVOS for all runs, order to produce a rough estimate of the upper bound on prediction accuracy. All runs were replicated under various perturbations to the model structure of MAFIA in order to assess the importance of various features in the model. We expect that the ability to easily perform such controlled experiments in the EVOS/MAFIA framework will enable end-users to test a wide array of both alignment algorithms and evolution models for regulatory sequence.

The first modification in the present experiment was to remove gain and loss states from the predictor’s model (while leaving them in the simulator’s model), producing what we call the “complete orthology” model. The next modification (called “Phylo-HMM”) employed a simple, three-state PPHMM for alignment, and then performed binding-site prediction by applying the full model (minus gain and loss states) to the root sequence; binding sites predicted in the root sequence were then mapped to leaf sequences via the homology relations implied by the predicted alignment. Note that the complete orthology model retains a potential advantage over our Phylo-HMM model: whereas the Phylo-HMM model performs alignment using a three-state background PHMM, the complete-orthology model performs alignment using the full model (minus gain and loss) and therefore incorporates knowledge of binding sites during alignment.

In addition to these modifications, we also applied a “single factors” model which utilized the full PPHMM with gain and loss states but only one of the seven factors used in the simulation runs; this was repeated for each factor and the binding-site predictions pooled for evaluation of binding-site prediction accuracy. Finally, we applied the full model with gain and loss states and all seven factors to a pruned version of the phylogeny containing only two species; for the first set of predictions we included *D. melanogaster* and *D. simulans*, while for the second set we used *D. melanogaster* and *D. mojavensis* (data from additional pairs are shown in Figure S2).


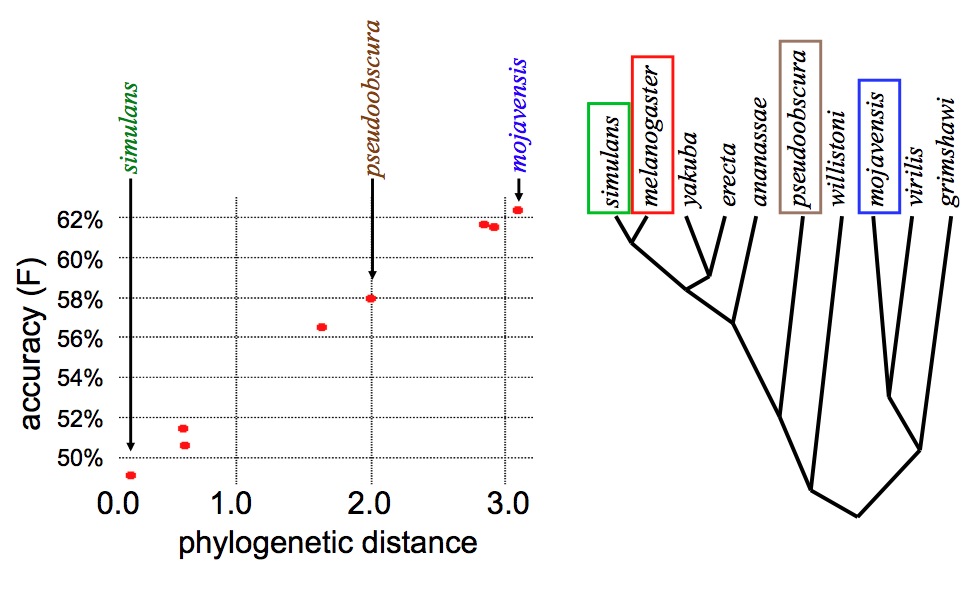


**Figure S2** : Site-level prediction accuracy (*F*-score), as a function of phylogenetic distance (sum of branch lengths) over a simulated *Drosophila* phylogeny (EVOS data set #2).

Results are shown in Table S2. As shown in the table, prediction accuracy (*F*-score) was drastically higher for the full model than for the complete-orthology model on data set #1, while the difference was much smaller for data set #2; this is consistent with the fact that the first data set has a lower average degree of orthology (52% versus 66%) and higher standard deviation (39% versus 14%), than the second set. The differences between the complete-orthology model and the Phylo-HMM were moderate but consistent, suggesting that the complete-orthology model does derive some advantage from its use of binding-site knowledge during alignment; recall that both models assume complete orthology during annotation, but only the “complete orthology” model includes states for binding sites during alignment. The single-factors model suffered from low specificity while enjoying high sensitivity, as expected, since this model utilized several runs of the predictor and was therefore able to predict overlapping binding sites for different factors.

|  | **data set #1** | | | | | | **data set #2** | | | | | |
| --- | --- | --- | --- | --- | --- | --- | --- | --- | --- | --- | --- | --- |
|  | **nucleotide** | | | **whole site** | | | **nucleotide** | | | **whole site** | | |
| **model** | **F** | **Sn** | **Sp** | **F** | **Sn** | **Sp** | **F** | **Sn** | **Sp** | **F** | **Sn** | **Sp** |
| full model | 41.2 | 35.0 | 50.2 | 39.5 | 34.8 | 45.7 | 81.5 | 71.4 | 95.0 | 83.8 | 76.8 | 92.2 |
| complete orthology | 25.7 | 47.0 | 17.0 | 19.3 | 38.8 | 12.8 | 81.2 | 85.9 | 77.0 | 80.4 | 90.9 | 72.1 |
| Phylo-HMM | 24.8 | 44.5 | 17.2 | 18.4 | 36.6 | 12.3 | 79.5 | 82.7 | 76.5 | 77.7 | 86.9 | 70.2 |
| single factors | 33.3 | 68.6 | 22.0 | 20.9 | 59.4 | 12.7 | 78.1 | 88.7 | 69.8 | 57.6 | 90.5 | 42.2 |
| dm3-moj3 | 38.0 | 26.0 | 70.2 | 38.8 | 27.3 | 66.9 | 58.8 | 42.1 | 97.5 | 62.3 | 46.2 | 95.4 |
| dm3-sim1 | 36.1 | 23.3 | 80.3 | 38.6 | 25.6 | 78.5 | 47.9 | 31.9 | 95.8 | 50.6 | 34.8 | 92.5 |

**Table S2** : Binding-site prediction accuracy for EVOS simulation runs. Left half: results from 108 simulations involving a phylogeny scaling factor in the range [0.01,90]. Right half: results from 100 simulations in which *λ* was varied from 0.001 to 0.01 while *μ* was varied from 0.01 to 0.1. Full model: 872-state model with gain and loss, 7 factors, 2 strands. Complete orthology: 132-state model lacking gain/loss states. Phylo-HMM: alignment with a 3-state model followed by annotation with the full model (872 states). Single factors: seven models, each for a single factor, with gain and loss. dm3-moj3: full model applied to 2-species phylogeny (*D. melanogaster*, *D. mojavensis*). dm3-sim1 : full model applied to 2-species phylogeny (*D. melanogaster*, *D. simulans*).

The pruning of the phylogeny to only two species had a much greater effect on the second data set, in terms of the absolute drop in accuracy. This may indicate that in highly divergent regimes (i.e., the first data set), accurate prediction is very difficult, and incorporating information from additional sequences adds nearly as much noise as signal. On the second data set it is also notable that the more divergent pair—*D. melanogaster* and *D. mojavensis*—produced substantially more accurate predictions, on average, than the much closer pair (total branch length: 2.9 versus 0.62), presumably indicating that for the second data set the superior contrast between foreground and background substitution rates is achieved at the larger evolutionary distance, whereas for the first data set, since branch lengths varied over nearly four orders of magnitude, the difference in combined branch lengths between the closer and further pairs of species is essentially of negligible effect. Figure S2 (above) plots the site-level accuracy as a function of phylogenetic distance on data set #2, from which a strong linear relationship can be clearly discerned.

Figure S3 plots the site-level prediction accuracy (*F*-measure) as a function of the average degree of orthology. The top two graphs correspond to the model incorporating cross-functional states for gain and loss of binding sites, while the bottom two graphs correspond to the model which assumes complete orthology. The left two graphs are for data set #1 and the right two graphs are for data set #2. On both data sets it is apparent that modeling gain and loss of binding sites produces a substantial increase in accuracy over a wide range of degrees of orthology. It is interesting to note that the complete-orthology model enjoys an advantage over the gain-loss model at the highest degree-of-orthology levels in data set #2, likely due to a higher sensitivity of the complete-orthology model in cases where sites do in fact exhibit complete orthology. However, the substantially greater correlation between accuracy and orthology levels for the complete orthology model on this data set (0.69, versus 0.46 for the full model) forces a rapid drop in prediction accuracy as orthology levels decrease.


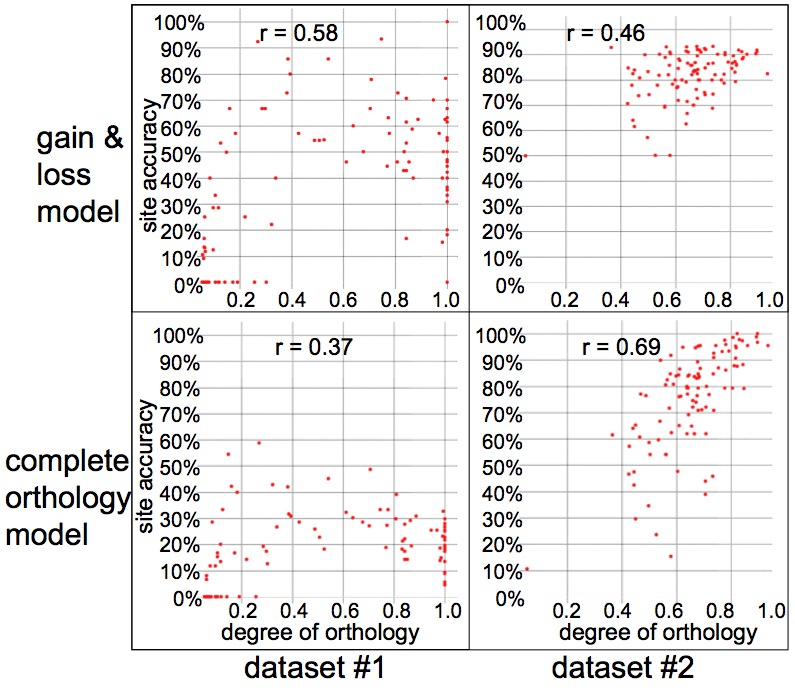


**Figure S3** : Site-level prediction accuracy (*F*-measure, *y*-axis) as a function of average degree of orthology (*x*-axis), for the complete-orthology model (top two graphs) and the full model (bottom two graphs), on data set #1 (left graphs) and data set #2 (right graphs). Correlation coefficients (*r*) are shown in each pane.

On data set #1 the degree-of-orthology was imposed more indirectly in the simulator, via scaling of the entire phylogeny. As a result, cases of high orthology generally occurred when the tree was scaled to have very small branch lengths, resulting in high conservation both in foreground and background regions and thus rendering the problem of discriminating regions under stronger selection (i.e., binding sites) much more difficult. This effect can be seen in the downward trend in both graphs above 60% orthology.

Table S3 shows the alignment accuracy for the EVOS simulation runs (both data sets pooled). Note that the alignment accuracy values for these runs were substantially lower than those for the PSPE simulations, likely due to the more varied parameterizations used in these simulations. While the advantage of the full model over the complete-orthology and Phylo-HMM models was extremely modest, it seems likely that any advantage which may be enjoyed over some limited simulation regimes is being obscured by averaging over such a large range of parameter values (particularly the scaling of the phylogeny).

| **model** | **F** | **SD** |
| --- | --- | --- |
| full model | 72.6 | 35.4 |
| complete orthology | 72.4 | 35.1 |
| phyloHMM | 72.2 | 35.4 |

**Table S3** : Average alignment accuracy for EVOS simulation runs; mean and SD values are given for both sets of simulation runs (pooled).

## **5.2 One Hundred Forty Two RedFly CRMs**

For this experiment we scored predictions based on whether they sufficiently overlap any part of a DNAse I footprint in the RedFly database. In order for a prediction to count as a true positive, we require that at least half of the predicted site overlap a RedFly footprint for the same factor. RedFly CRMs are likely to contain many other valid binding sites of the same factor in addition to those evidenced by a footprint, and assessing false positive rate of predictions on these regions is problematic. To address this, we additionally evaluate false positive rate on a set of appropriate decoy CRMs selected randomly from the genome.

We chose transcription factors for which (1) each factor had a JASPAR matrix deriving from bacterial 1-hybrid evidence (Noyes *et al.*, 2008), (2) each factor came from a different protein family, and (3) at least 20 RedFly CRMs were available for each factor. The first requirement eliminates the potential for circularity as mentioned above, as the matrices were derived from independent experiments. This resulted in three factors (Ubx, tll, Kr) and a total of 142 CRMs. For each of these true CRMs we randomly selected a 500bp interval of approximately equal G+C content and PhastCons (Siepel *et al.*, 2005) conservation score to serve as a “decoy” CRM. The phylogeny used for this experiment was (*virilis,* (*pseudoobscura,* (*ananassae,* (*melanogaster,* (*yakuba*, *erecta*))))), and we assumed that UCSC whole-genome alignments were accurate enough to correctly identify orthologous regions.

Rather than comparing against a set of “competing” approaches run on default parameters, we chose our evaluations to focus on specific previous methods run on task-adjusted parameters, which allowed us to fairly assess the impact of different model assumptions. Model parameters for MAFIA were estimated on the comparatively well-annotated segmentation enhancers (see below), and we chose rMONKEY for comparison as it allows evolutionary site inference on multiple related species. Instead of simply running rMONKEY on genomic alignments which might be problematic, we provided it with sequences realigned by MUSCLE, as we had observed MUSCLE to achieve the same nucleotide level alignment accuracy as our approach. We then selected rMONKEY’s significance threshold so as to produce the same average false positive rate (i.e., the same specificity) as our program on the decoy sites.

|  |  | **predictions per kilobase** | |
| --- | --- | --- | --- |
| **program** | **Sn** | **in true CRMs** | **in decoy CRMs** |
| MAFIA | 52.2 | 21.4 | 12.9 |
| rMONKEY | 50.3 | 20.7 | 12.9 |

**Table S4** : Sensitivity of binding site predictions on 142 RedFly CRMs. Number of predictions per kilobase of sequence are in *D. melanogaster* only. Decoy CRMs were sampled from the genome to have approximately identical G+C content and PhastCons conservation levels as the RedFly CRMs.

As shown in Table S4, MAFIA achieved a higher sensitivity than rMONKEY in RedFly CRMs when controlling for false positive rate in decoy CRMs, resulting in a proportional reduction in false negative rate of 3.8%. These results mirrored the simulations above; the simpler rMONKEY model lead to a competitive sensitivity, but anecdotal observations revealed that this was partially based on incorrect orthologue predictions in the non-target species (data not shown). Both programs predict roughly 60% more binding sites in true RedFly CRMs than in decoy CRMs of the same G+C content and conservation level, demonstrating that the RedFly CRMs are indeed likely to contain additional non-annotated true positives. Decoy sites were chosen genome-wide to have G+C and conservation levels matching RedFly enhancers, and some of the decoy CRMs may thus in fact overlap regulatory regions and may overestimate the false positive rate. However, for the purpose of comparing sensitivities of the two predictors at the same specificity level, we believed this to be a useful protocol.

## **5.3 Seventeen RedFly CRMs**

Table S5 gives the confusion matrix for predicted versus known sites in the “gold standard” for the 17 *Drosophila* CRMs. Values along the diagonal indicate percentages of our predictions that overlapped a known site of the same factor (by at least 1bp). Off-diagonal entries indicate overlap between predictions for one type of factor and known sites for another factor.

|  | **cad** | **kni** | **gt** | **Kr** | **bcd** | **hb** | **tll** | **totals** |
| --- | --- | --- | --- | --- | --- | --- | --- | --- |
| **cad** | 0.2 | 0 | 0 | 0 | 0 | 0 | 0 | 5 |
| **kni** | 0.06 | 0.18 | 0 | 0 | 0.06 | 0 | 0 | 17 |
| **gt** | 0 | 0 | 0.4 | 0 | 0.2 | 0.1 | 0.1 | 10 |
| **Kr** | 0.04 | 0 | 0 | 0.19 | 0.04 | 0 | 0.08 | 26 |
| **bcd** | 0 | 0 | 0 | 0.09 | 0.59 | 0 | 0 | 22 |
| **hb** | 0.01 | 0.08 | 0.01 | 0.08 | 0.06 | 0.42 | 0.05 | 79 |
| **tll** | 0 | 0 | 0 | 0.1 | 0 | 0 | 0.2 | 10 |

**Table S5** : Confusion matrix for binding sites in 17 *Drosophila* CRMs. Rows correspond to predicted sites; columns correspond to known sites in the “gold standard”. Decimal numbers in the interior of the table give the percentage of predicted sites of each type that overlapped by at least 1bp with known sites of each type. The “totals” column gives absolute counts of predicted sites of each factor.

Table S6 gives the overlap matrix for known sites in the “gold standard” for the 17 *Drosophila* CRMs. Off-diagonal entries indicate overlaps between known sites of different factors.

|  | **cad** | **kni** | **gt** | **Kr** | **bcd** | **hb** | **tll** | **totals** |
| --- | --- | --- | --- | --- | --- | --- | --- | --- |
| **cad** | 0 | 0 | 0 | 0 | 0.29 | 0 | 0 | 7 |
| **kni** | 0 | 0 | 0 | 0 | 0.12 | 0.27 | 0.12 | 26 |
| **gt** | 0 | 0 | 0 | 0 | 0.2 | 0.2 | 0 | 5 |
| **Kr** | 0 | 0 | 0 | 0.07 | 0.14 | 0.1 | 0.04 | 29 |
| **bcd** | 0.06 | 0.09 | 0.03 | 0.12 | 0 | 0.09 | 0.09 | 34 |
| **hb** | 0 | 0.14 | 0.02 | 0.06 | 0.06 | 0 | 0.02 | 49 |
| **tll** | 0 | 0.16 | 0 | 0.05 | 0.16 | 0.05 | 0 | 19 |

**Table S6** : Overlap matrix for known binding sites 17 *Drosophila* CRMs. Values in the interior of the matrix give the percentage of sites of each type that overlapped (by at least 1bp) another site of a different factor.

We now provide several additional example alignments and binding-site annotations produced via MAFIA. Figure S4 shows that our system is able to predict sites even when insertions and/or deletions are present in the resulting alignment. This ability is due to the rows of insertion and deletion states shown in Figure 2 (main article). These additional indel states result in a rapid growth of model size as factors are incorporated into the model, rendering our use of the Hirschberg algorithm for decoding essential when more than a few (double-stranded) factors are included in the model.


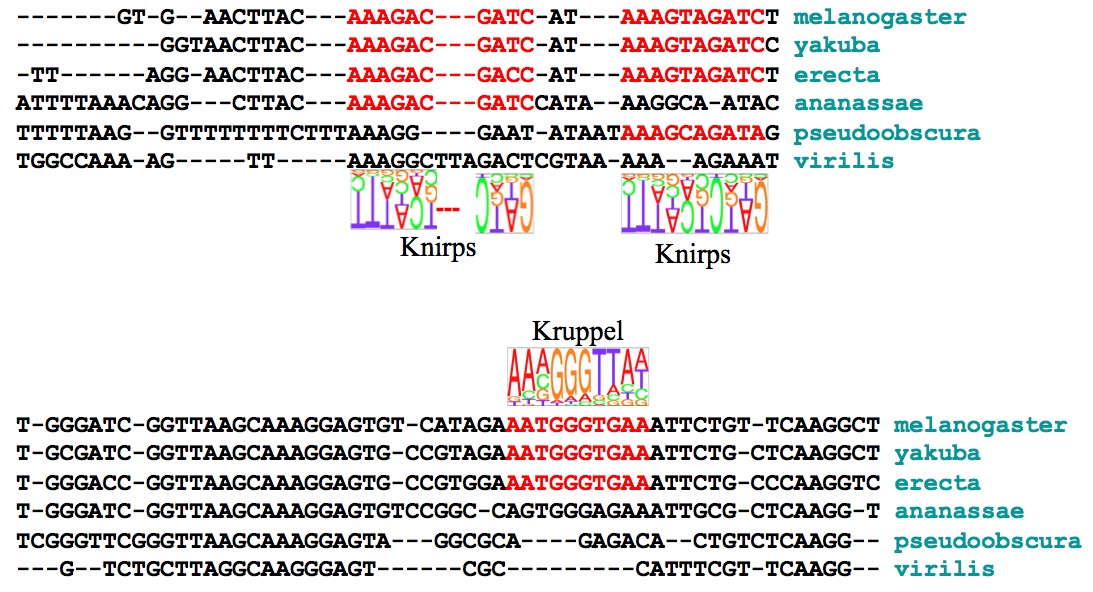


**Figure S4** : Examples of indels in non-functional sequences orthologous to putative binding sites. Alignments and annotations were produced by MAFIA. Red nucleotides are predicted to be functional, black nonfunctional.

Figure S5 depicts two additional examples involving functional turnover, one in a 6-way alignment and the other for a 10-way alignment. As noted earlier, since the computational complexity of our alignment algorithm grows only linearly with the number of sequences, we are able to simultaneously align and annotate CRMs from an arbitrary number of species, a challenge for many other systems.


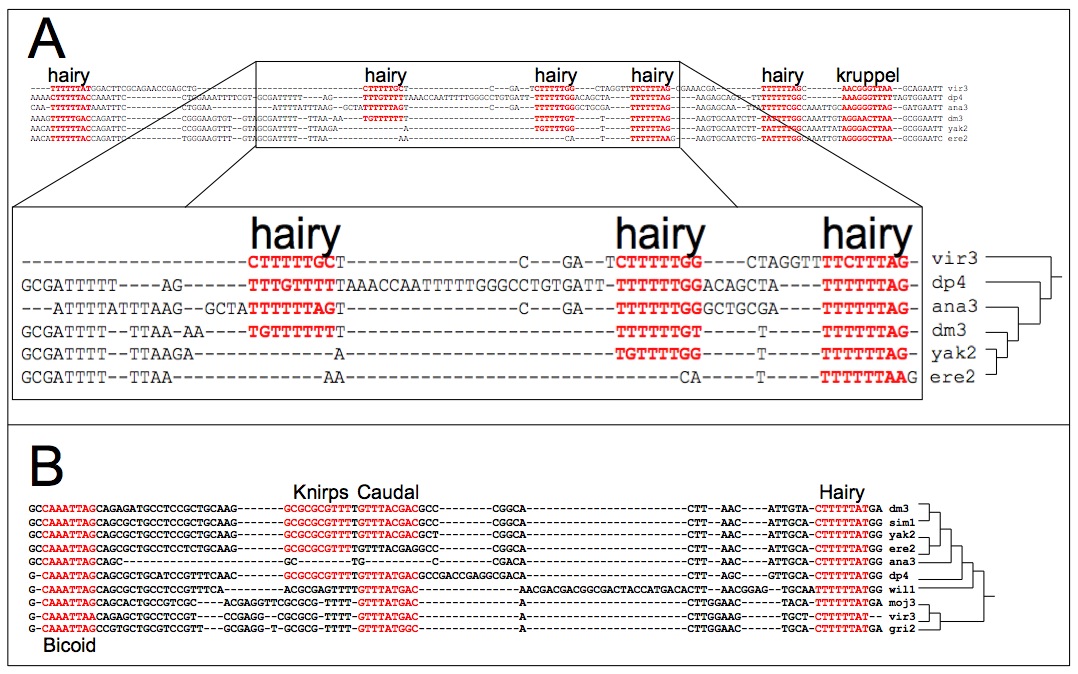


**Figure S5** : (a) Example of predicted loss events in a region rich with putative binding sites for the same factor (*hairy*). (b) Another example involving functional turnover, this time in the context of a 10-way alignment.

The existence of redundant, proximal, putative sites for the same factor may to some extent relax selective constraints on individual sites within a CRM. In part (a) of the figure, aligned blocks with strong sequence identity flanking the sites provide support to the hypothesis that these sites are indeed absent from the respective taxa (*D. yakuba* and *D. erecta* for the leftmost site in the zoomed pane, and *D. erecta* in the middle site). This example illustrates both the utility of cross-functional states in our model and the potential for further extensions to our framework, since we currently do not capture compensatory relations between spatially clustered sites, and/or selection on the level of a whole CRM; to our knowledge, no other comparable system captures such relations, though the need to do so is abundantly clear.

# 6. Binding-site Profiles

## **6.1 Weight Matrices from JASPAR**

The following JASPAR positional weight matrices were used in the RedFly computational experiments.

*1. Ubx (JASPAR: MA0094.2)*


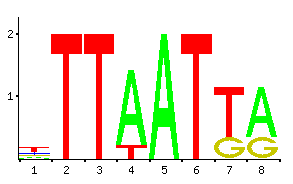


*2. Kr (JASPAR: MA0452.1)*


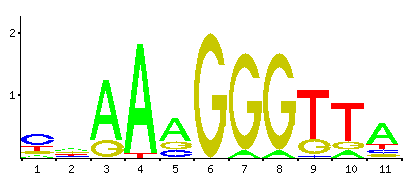


*3. tll (JASPAR: MA0459.1)*


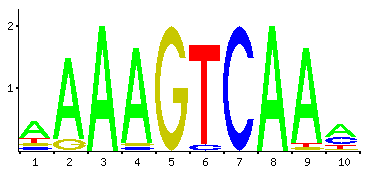


## **6.2 Weight Matrices from EMMA Package**

The following positional weight matrices were obtained from the EMMA package and were used in the 17-CRM comparisons.

*1. bcd (bicoid)*


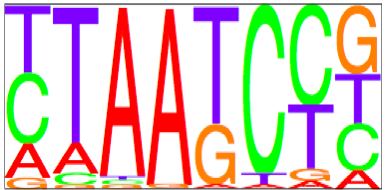


*2. cad (caudal)*


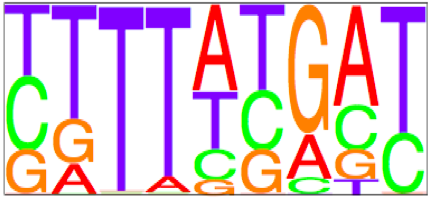


*3. gt (giant)*


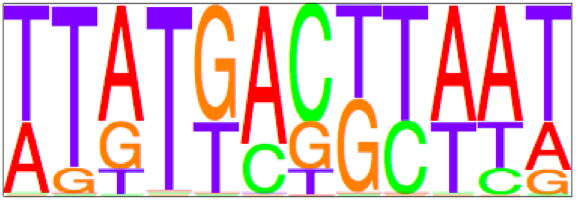


*4. hb (hunchback)*


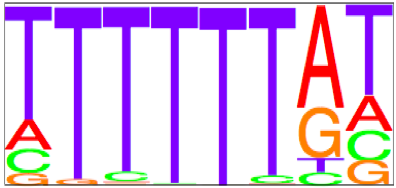


*5. kni (knirps)*


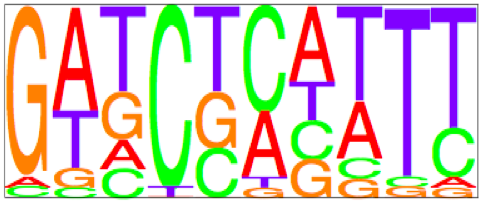


*6. Kr (kruppel)*


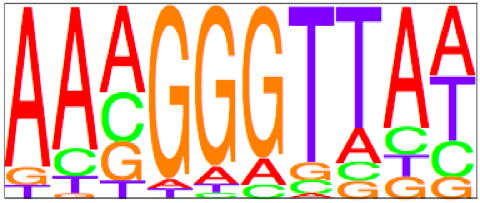


*7. tll (tailless)*


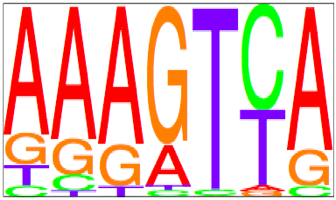


# References

Boyle AP, Guinney J, Crawford GE, Furey TS (2008) F-Seq: a feature density estimator for high-throughput sequence tags. *Bioinformatics* 24:2537–2538.

Bradley RK, Li X-Y, Trapnell C, Davidson S, Pachter L, *et al.* (2010) Binding Site Turnover Produces Pervasive Quantitative Changes in Transcription Factor Binding between Closely Related Drosophila Species. *PLoS Biol* 8:e1000343.

Bryne JC, Valen E, Tang MH, Marstrand T, Winther O, da Piedade I, Krogh A, Lenhard B, Sandelin A (2008) JASPAR, the open access database of transcription factor-binding profiles: new content and tools in the 2008 update. *Nucleic Acids Res* 36:D102-6.

Church A (1932) A set of postulates for the foundation of logic. *Ann Math, Series 2*, 33:346–366.

Durbin R, Eddy S, Krogh A, Mitchison G (1998) *Biological Sequence Analysis*. Cambridge University Press.

Edgar RC (2004) MUSCLE: multiple sequence alignment with high accuracy and high throughput. *Nucleic Acids Res* 32:1792-97.

Felsenstein J (1981) Evolutionary trees from DNA sequences. *J Mol Evol* 17:368-376.

Gumucio DL, Heilstedt-Williamson H, Gray TA, Tarlé SA, Shelton DA, Tagle DA, Slightom JL, Goodman M, Collins FS (1992) Phylogenetic footprinting reveals a nuclear protein which binds to silencer sequences in the human gamma and epsilon globin genes. *Mol Cell Biol* 12:4919-29.

Halfon MS, Gallo SM, Bergman CM (2008) REDfly 2.0: an integrated database of cis-regulatory modules and transcription factor binding sites in Drosophila. *Nucleic Acids Res* 36:D594-598.

Halpern AL, Bruno WJ (1998) Evolutionary distances for protein-coding sequences: modeling site-specific residue frequencies. *Mol Biol Evol* 15:910-917.

Hawkins J, Grant C, Noble WS, Bailey TL (2009) Assessing phylogenetic motif models for predicting transcription factor binding sites. *Bioinformatics* 12:i339-i347.

He X, Ling S, Sinha S (2009). Alignment and Prediction of cis-Regulatory Modules Based on a Probabilistic Model of Evolution. *PLoS Comput Biol* 5:e1000299.

Hirschberg, DS (1975) A linear space algorithm for computing maximal common subsequences. *Commun ACM* 18:341-343.

Holmes I, Bruno WJ (2001) Evolutionary HMMs: a Bayesian approach to multiple alignment. *Bioinformatics* 17:803-820.

Huang W, Nevins JR, Ohler U (2007) Phylogenetic simulation of promoter evolution: estimation and modeling of binding site turnover events and assessment of their impact on alignment tools. *Genome Biol* 8:R225.

Langmead B, Trapnell C, Pop M, Salzberg SL (2009) Ultrafast and memory-efficient alignment of short DNA sequences to the human genome. *Genome Biol.* 10:R25.

Loytynoja A, Goldman N (2008) Phylogeny-aware gap placement prevents errors in sequence alignment and evolutionary analysis. *Science* 320:1632-1635.

Ludwig MZ, Kreitman M (1995) Evolutionary dynamics of the enhancer region of even-skipped in Drosophila. *Mol Biol Evol* 12:1002-1011.

Ludwig MZ, Palsson A, Alekseeva E, Bergman CM, Nathan J, *et al.* (2005) Functional evolution of a cis regulatory module. *PLoS Biol* 3:e93.

Lusk RW, Eisen MB (2010) Evolutionary mirages: selection on binding site composition creates the illusion of conserved grammars in Drosophila enhancers. *PLoS Genet* 6:e1000829.

Majoros WH (2007) *Methods for Computational Gene Prediction*. Cambridge University Press.

Majoros WH, Ohler U (2009) Complexity reduction in context-dependent DNA substitution models. *Bioinformatics* 25:175-182.

Moses AM, Pollard DA, Nix DA, Iyer VN, Li XY, Biggin MD, Eisen MB. (2006) Large-scale turnover of functional transcription factor binding sites in Drosophila. *PLoS Comput Biol* 2:e130.

Noyes MB, Christensen RG, Wakabayashi A, Stormo GD, Brodsky MH, Wolfe SA (2008) Analysis of homeodomain specificities allows the family-wide prediction of preferred recognition sites. *Cell* 133:1277-89.

Quesne WJL (1974) The uniquely evolved character concept and its cladistic application. *Syst Zool* 23:513-517.

Pollard DA, Moses AM, Iyer VN, Eisen MB (2006) Detecting the limits of regulatory element conservation and divergence estimation using pairwise and multiple alignments.  *BMC Bioinformatics* 7:376.

Ray PR, Shringarpure S, Kolar M, Xing EP (2008) CSMET: Comparative genomic motif detection via multi-resolution phylogenetic shadowing. *PLoS Comput Biol* 4:e1000090.

Saitou N, Nei M (1987) The neighbor-joining method: a new method for reconstructing phylogenetic trees. *Mol Biol Evol* 4:406-425.

Satija R, Pachter L, Hein J (2008) Combining statistical alignment and phylogenetic footprinting to detect regulatory elements. *Bioinformatics* 24:1236-1242.

Siddharthan R (2008) PhyloGibbs-MP: Module Prediction and Discriminative Motif-Finding by Gibbs Sampling. *PLoS Computat Biol* 4(8) e1000156. doi:10.1371/journal.pcbi.1000156.

Siepel A, Bejerano G, Pedersen JS, Hinrichs AS, Hou MM, Rosenbloom K, Clawson H, Spieth J, Hillier LW, Richards S, *et al.* (2005) Evolutionarily conserved elements in vertebrate, insect, worm, and yeast genomes. *Genome Res* 15:1034-1050.

Siepel A, Haussler D (2004) Combining phylogenetic and hidden Markov models in biosequence analysis. *J Comput Biol* 11:413-428

Thompson JD, Higgins DG, Gibson TJ (1994) CLUSTAL W: improving the sensitivity of progressive multiple sequence alignment through sequence weighting, position-specific gap penalties and weight matrix choice. *Nucleic Acids Res* 22:4673-4680.

Thorne JL, Kishino H, Felsenstein J (1991) An evolutionary model for maximum likelihood alignment of DNA sequences. *J Mol Evol* 33:114-124.

Viterbi A (1967) Error bounds for convolutional codes and an assymptotically optimal decoding algorithm. *IEEE T Inform Theory* IT-13:260-269.

Wagner GP, Otto W, Lynch V, Stadler PF (2007) A stochastic model for the evolution of transcription factor binding site abundance. *J Theor Biol* 247:544-553.

Wong K, Suchard M, Huelsenbeck J (2008) Alignment uncertainty and genomic analysis. *Science* 319:473-476.

Wray G (2007) The evolutionary significance of cis-regulatory mutations. *Nat Rev Genet* 8:206-216.
